# Production of Food-Derived Bioactive Peptides with Potential Application in the Management of Diabetes and Obesity: A Review

**DOI:** 10.1021/acs.jafc.2c08835

**Published:** 2023-04-07

**Authors:** Weiwei Wang, Wenjian Yang, Yi Dai, Jianhui Liu, Zhen-Yu Chen

**Affiliations:** † College of Food Science and Engineering, Nanjing University of Finance and Economics/Collaborative Innovation Center for Modern Grain Circulation and Safety, Nanjing 210023, China; ‡ Food & Nutritional Sciences Programme, School of Life Sciences, The Chinese University of Hong Kong, Shatin, NT, Hong Kong, China

**Keywords:** peptides, enzymes, diabetes, obesity, inflammation

## Abstract

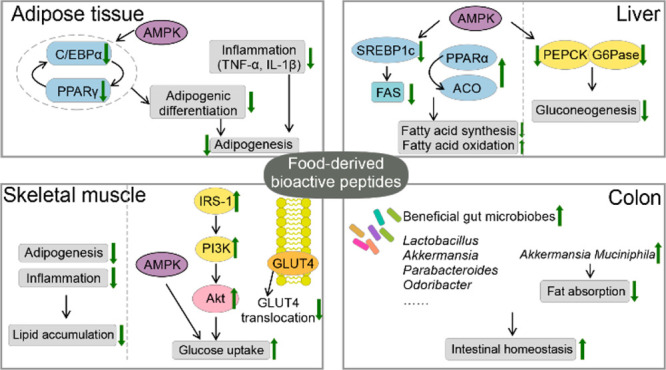

The prevalence of diabetes mellitus and obesity is increasing worldwide. Bioactive peptides are naturally present in foods or in food-derived proteins. Recent research has shown that these bioactive peptides have an array of possible health benefits in the management of diabetes and obesity. First, this review will summarize the top-down and bottom-up production methods of the bioactive peptides from different protein sources. Second, the digestibility, bioavailability, and metabolic fate of the bioactive peptides are discussed. Last, the present review will discuss and explore the mechanisms by which these bioactive peptides help against obesity and diabetes based on *in vitro* and *in vivo* studies. Although several clinical studies have demonstrated that bioactive peptides are beneficial in alleviating diabetes and obesity, more double-blind randomized controlled trials are needed in the future. This review has provided novel insights into the potential of food-derived bioactive peptides as functional foods or nutraceuticals to manage obesity and diabetes.

## Introduction

Diabetes mellitus (DM) and obesity are two chronic conditions related to metabolic syndrome, and their prevalence is increasing globally.^1,2^ Obesity is caused by an increase in body weight and an accumulation of excess body fat. Numerous studies have shown that obesity is a main risk factor for type 2 diabetes mellitus (T2D).^3^ DM is characterized by having an elevated blood glucose level resulting from inadequate insulin secretion and/or its action. DM falls into two categories: type I and type II. In brief, type I diabetes, namely, insulin dependent DM, is an autoimmune disorder characterized by dysfunction of beta cells and little or no insulin secretion by the pancreas. T2D, also called noninsulin dependent DM, involves an imbalance in insulin production and blood glucose uptake.^4^ Inadequate treatments of DM can result in serious complications, such as cardiovascular diseases, nephropathia, diabetic retinopathy, amputation, and nerve damage.^5^ Both obesity and DM are strongly associated with an unhealthy diet such as a high-fat diet (HFD) and a high-sugar carbohydrate diet for a long time. In addition to antidiabetic and antiobesity medications, it is known that some bioactive ingredients from natural foods are beneficial in the management of obesity and DM.

Bioactive peptides are naturally present in foods or in food-derived proteins due to the partial hydrolysis of proteins during digestion or processing. Normally, they contain a free carboxyl and a free amino group. Some exception is seen in recent studies demonstrating that, after enzymatic hydrolyses, peptides can have no free amino group such as prolyl and pyroglutamyl peptides.^6^ Recently, research has shown that a large number of bioactive peptides from food proteins have an array of biological functions including antimicrobial, anticancer, antiobesity, as well as antidiabetes effects. As a dietary supplement or nutraceutical, these peptides provide a new strategy to manage the chronic diseases.^7^ Different from intact proteins, these bioactive peptides commonly have a low molecular weight and thus have better digestibility and bioavailability.^8^ In the present review, the top-down and bottom-up production methods and bioavailability of bioactive peptides from different food sources are summarized. The possible molecular actions of food-derived bioactive peptides against obesity and diabetes are explored to elucidate the underlying mechanisms.

## Production, Purification, and Characterization of Bioactive Peptides

### Production

**Figure 1:**
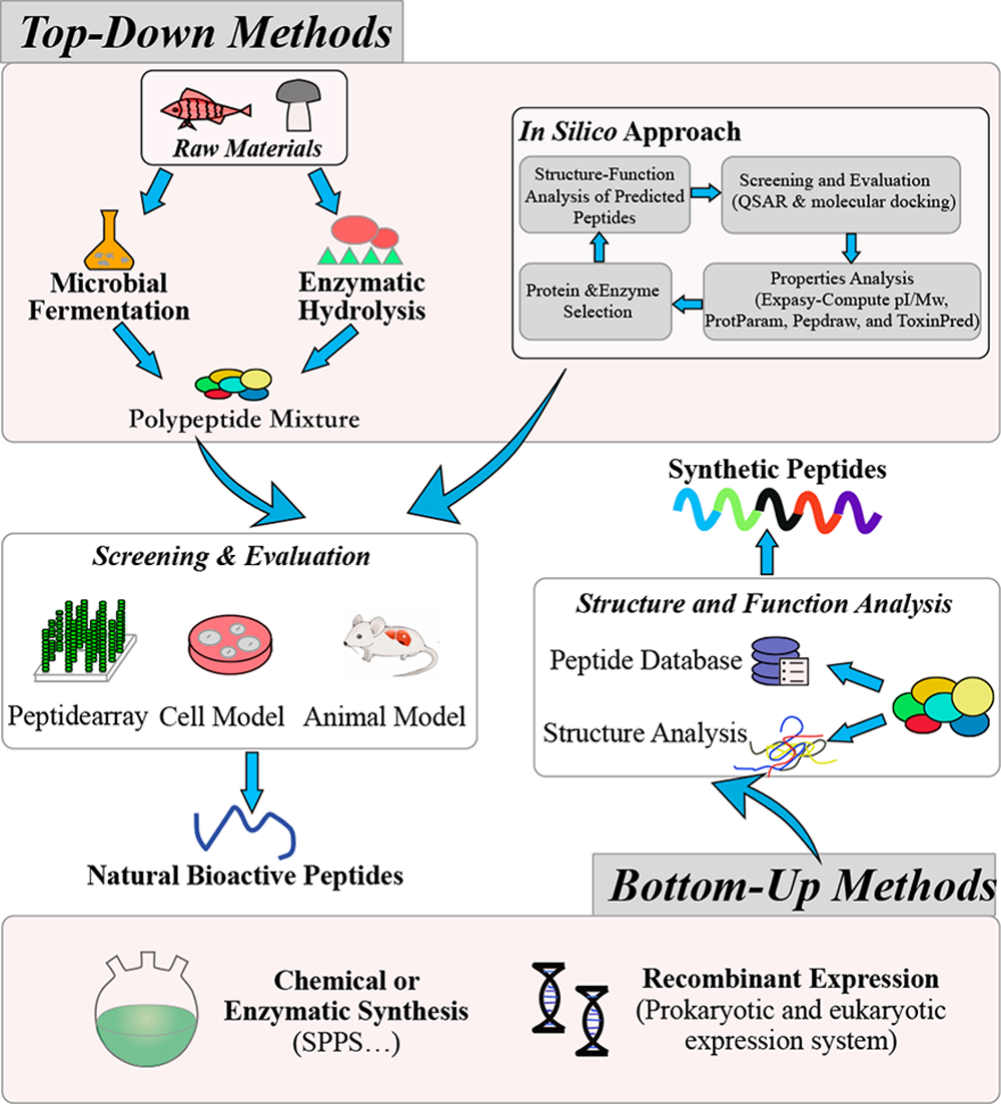
Production of bioactive peptides by top-down and bottom-up methods. Top-down refers to the process of preparation, separating, and purifying raw food-originated materials to obtain the bioactive peptides, mainly including the microbial fermentation and enzymatic hydrolysis. The *in silico* approach is an emerging top-down method based on bioinformatics to predict the cleavage site of a protein sequence with a specific protease under some given conditions. Bottom-up method refers to the synthesis of a specific polypeptide sequence from amino acids, mainly including chemical or enzymatic synthesis and recombinant expression. QSAR, quantitative structure–activity relationships; SPPS, solid-phase peptide synthesis.

Food-derived bioactive peptides mainly originate from the partial hydrolysis of proteins derived from plants, animals, and edible fungi. The existing production technologies of bioactive peptides can be divided into two categories: top-down and bottom-up (Figure 1). Top-down method mainly refers to the process of separating and purifying the raw protein materials to obtain bioactive polypeptides, mainly including enzymatic hydrolysis, microbial fermentation, and *in silico* proteolysis.^2,9−12^ The bottom-up method refers to the process of synthesizing specific polypeptide sequences from amino acids, mainly including chemical or enzymatic synthesis or genetic recombination.^13^ The premise of this method is that the amino acid sequence is known. These two production methods and sources of bioactive peptides are summarized in Table 1.

**Table 1:** Production Methods and Protein Sources of Bioactive Peptides

peptides (sequence)	source	production methods	parent protein	reference
Top-Down				
YINQMPQKSRE, YINQMPQKSREA, VTGRFAGHPAAQ	Egg yolk	Pepsin (120 min)	/	(44)
Ovotransferrin: RVPSLM, TPSPR, DLQGK, AGLAPY	Egg white	Alcalase (180 min)	Ovotransferrin	(45)
Ovalbumin: RVPSL, DHPFLF HAGN, WIGLF			Ovalbumin	
LPDEVSG DDNKVED, GVDTKSD, IESGSVEQA GGLVVT	Egg white	Peptidase (24 h)	/	(46)
FRADHPPL, FSL, SALAM, YQIGL, RADHPFL, IVF, YAEERYPIL, YRGGLEPINF, RDILNQ, ESIINF	Egg white	Pepsin (8 h)	/	(46)
IRW, IQW, LPK	Egg white	Thermolysin-pepsin (6 h)	Ovotransferrin	(47)
AEERYP, DEDTQAMP	Egg white	Protease (3 h)	/	(15)
VPP	Calpis sour milk	Protease (14 h)	Casein	(48)
VLPVPQK	Buffalo milk	Trypsin-pepsin (6 h)	Casein	(49)
AQSAP, IPAVF, APLRV, AHKAL	Bovine milk	Fermentation (36 h) using Lactobacillus and Helvelticus	Whey	(50)
PGPIPN, PFPGPIPN, YPFPGPIP, VYPFPGPIPN, MPFPKYPVEP, EPVLGPVRGPFP, QEPVLG, PVRGPFP, TPVVVPPFLQPE, TQTPVVVPPFLQPE	Bovine milk	Favorzyme, savinase, thermolysin, trypsin, and elastase (45 min)	Casein	(16)
DPYKLRP, PYKLRP, YKLRP, GILRP	Lactoferrin	Fermentation using Kluyveromyces and Marxianus	Lactoferrin	(51)
LPVPGTL	Camel milk	Trypsin (2.5 h)	/	(52)
ELLLNPTHQIYPVTQPLAPV, AMPSSSEESII	Human milk	/	Casein 201–220 aa	(53)
YQEPVLGPVRGPFP, FVAPFPEVF, PPFLQPEVM, QEPVLGPVRGPFPIIV	Yogurt	Pepsin, trypsin, chymotrypsin, elastase and carboxypeptidase A (1 h)	β-casein, αS1-casein	(54)
IPP, VPP	Fermented milk	Lactobacillus, Saccharomyces	β-casein	(55)
LRFF	Fermented milk	*Kluyveromyces marxianus*	κ-casein	(56)
VLSRYP			αS1-casein	
FVAPEPFVFGKEK	Fermented milk	Kombucha culture (48 h)	β-casein	(57)
LVYPFPGPLH			αS1-casein	
VAPFPEVFGK			αS2-casein	
				
LVESPPELNTVQ, VLESPPELN, WGYLAYGLD	Fermented milk	Lactobacillus casein (48 h)	κ-casein	(57)
INNQFLPYPY	Goat (*Capra hircus*) milk	Trypsin (3 h)	κ-casein	(58)
MHQPPQPL			β-casein	
LDQWLCEKL	Whey proteins	Trypsin (1 h)	α-Lactalbumin	(59)
GKFNV, LPGGGHGDL	Chinese Jinhua dry-cured ham	Pepsin (2 h) and trypsin (2 h)	/	(60)
DLEE	Chinese Xuanwei dry-cured ham	Fermentation (8 months)	/	(61)
GVVPL, LGL, SFVTT	Italian Parma dry-cured ham	*In vitro* gastrointestinal digestion for 18 and 24 months	/	(62)
FNMPLTIRITPGSKA	Spanish dry-cured ham	Water extraction at 4 °C	LIM domain-binding 3	(63)
HCNKKYRSEM	Spanish dry-cured ham	Reversed-phase HPLC separation	Dynein heavy chain	(64)
MDPKYR, TKYRVP			Titin	
SNAAC	Spanish dry-cured ham	Water extraction at 4 °C	Myosin heavy chain	(65)
AAATP, TSNRYHSYPWG	Spanish dry-cured ham	Water extraction at 4 °C	Allantoicase	(66)
MWTD, APYMM, FWIIE	Chinese dry-cured Mmtton ham	Water extraction	/	(67)
AVPYPQ, EAMAPK	Italian Stracchino cheese	Pepsin (2 h)	β-casein	(68)
RPKHPIKHQ, RPKHPIKHQG	Brazilian Canastra artisanal Minas cheese	Water extraction	αS1-casein	(69)
DKIHPF	Hard cow milk cheese	Water extraction	β-casein	(70)
EIVPN, VAPFPQ			αS1-casein	
YVEELKPTPEGDL	Buffalo ricotta cheese	Simulated gastrointestinal digestion using pepsin, pancreatin and chymotrypsin (2 h)	/	(71)
QEPVLGPVRGPFPIIV	Brazilian Prato cheese	Water extraction at pH 4.6 and 70% ethanol	β-casein	(72)
APFPE	Parmigiano-Reggiano cheese	Ultrafiltration	/	(73)
IF, SV, WP	Fermented shrimp pastes	Fermentation (6 months)	/	(74)
Marine collagen peptides	Fish	Mixed proteases (pepsin, trypsin, chymotrypsin, and pancreatic lipase) for 3 h	Marine collagen	(18)
AIPPHPYP, FF	Fish	*Malaysian pekasam*/*Lactobacillus plantarum*	/	(24)
GPAV	*Atlantic salmon*	Corolase PP (1 h)	/	(75)
GLSRLFTALK	*Atlantic mackerel*	Protamex (24 h)	/	(76)
NAPNPR, YACSVR	*Sardine pilchardus* protein	Subtilisin	/	(77)
AFVGYVLP, EKSYELP, VELYP	Cuttlefish	*Bacillus mojavensi* (pH 10.0; 50 °C); cuttlefish hepatopancreas enzymes (pH 8.0, 50 °C)	/	(78)
AER, EQIDNLQ	Leatherjacket	Insoluble bromelain (2 h)	/	(79)
DPHI, EPLYV	Leatherjacket	Insoluble papain (6 h)	/	
ITALAPSTM, SLEAQAEKY, GTEDELDKY	Sardinelle	*Bacillus subtilis* A26, *B. amyloliquefaciens* An6 (24h)	Actin, Tropomyosin	(80)
NVPVYEGY		*Bacillus subtilis*	Actin	
KEEKFE, LHDELT	*Pacific herring*	Trypsin (7 h)	/	
SY	Jelly fish gonads	Pancreatin (2.5 h)	/	(81)
Collagen peptide	Walleye pollock	Flavourzyme (2 h), then Alcalase (2 h), and trypsin (2 h)	/	
AFMNVKHWPW, SFMNVKHWPW, WPW	Spent hens	Protex 50FP (3 h)	Myosin	(82)
FLWGKSY			Myomesin	
AGRDLTDYLMKIL, GYDLGEAEFARIM, IEDPFDQDDWGAWKK, LQAEVEELRAALE, NWDDMEK	Duck (*Anas platyrhynchos)*	Protamex (4 h)	/	(83)
FQPS	*Kacang goat (Capra aegagrus hircus)*	Protamex + Flavourzyme (4 h)	Actin	(84)
LVGRPRHGQ, VFPS	Pork loin	Thermolysin (24 h)	/	(85)
AAATP	Porcine skeletal muscle protein	Meat-borne Lactobacillus fermentation (96h)	/	(25)
APGLPGPR, FPGPPGP, Piro-GPPGPT	Chicken combs and wattles	Alcalase (5%, 4 h)	Collagen and elastin	(86)
APGAPGPVG, HYVPV	Chicken hydrolysates (collagen)	Simulated gastrointestinal digestion using pepsin, trypsin and chymotrypsin (37 °C, 2 h)	Collagen alpha-1(I) chain	(87)
WG	Poultry protein	Simulated gastrointestinal digestion using pepsin trypsin and chymotrypsin (37 °C, 2 h)	Hemoglobin subunit alpha-β	(87)
NALKCCHSCPA, LNNPSVCDCDCMMKAAR, NPVWKRK, CANPHELPNK	*Spirulina platensis*	Five enzymes (trypsin, alcalase, pepsin, papain and protamex) for 10 h	/	(88)
IAVPTGVA, YVVNPDNDEN, YVVNPDNNEN	Soy	Digestion with trypsin or pepsin	/	(89)
ILL, LLL, VHVV	Soy	Flavourzyme (125 min)	/	(90)
IAVPGEVA, IAVPTGVA, LPYP	Soy	Trypsin or pepsin	/	(91)
Aglycin (37 aa)	Soy	Hydrolysis in 60% methanol (pH 4.8)	/	(92)
LPTPA	Soybean	Trypsin (4 h, pH 8.0)	Soybean glycinin A_5_A_4_B_3_ subunit	(93)
YVVFK	Soybean	Simulated gastrointestinal digestion using pepsin (1 h) and pancreatin (2 h)	Soybean seeds and soy milk protein	(94)
LPYP, IAVPGEVA, IAVPTGVA	Soybean protein	Pepsin (2–24h)	Soy glycinin	(95)
RNLQGENEEEDSGA	Germinated soybean	Simulated gastrointestinal digestion using pepsin (1 h) and pancreatin (2 h)	Germinated soybean protein	(96)
VVSLSIPR	Pigeon pea seeds	*Aspergillus niger* at 27 °C for 7 days	/	(97)
YAAAT	Black bean and cowpea	Pepsin-pancreatin (pH 7.0)	/	(98)
LLSGTQNQPSFLSGF, NSLTLPILRYL, TLEPNSVFLPVLLH	Lentil protein	Savinase at 40 °C for 2 h	/	(99)
LAYLQYTDFETR	Pecan	Alcalase (3 h)	Pecan protein	(18)
Antioxidant Peptides	Carrot seeds	Four proteases (papain, trypsin, Neutrase, and alcalase) for 3.5 h	Carrot seed protein	(100)
HSDADYVLVVLNGR, HGREEEEEEEEEDER, YPSSTKDQQSY	White lupin seed	Pepsin and trypsin (18 h)	/	(101)
YVDGSGTPLT, PHPATSGGGL, and GKAAPGSGGGTKA	Chickpea protein	A simulated digestive system (0.5 h) (pepsin/pancreatin)	/	(102)
WVYY, PSLPA	Hemp seed	Simulated gastrointestinal digestion using pepsin (2 h) and pancreatin (4 h)	/	(103)
YSK	Rice bran	Trypsin (2 h)	/	(104)
DVWY, FQ, VVG, VAE, WTFR	Buckwheat sprout	Neo-fermentation (2 weeks)	/	(105)
FPFPPTLGY and FPFPR	Amaranth proteins	Bromelain (2 h), chymotrypsin (2 h), and Pronase E (2 h)	/	(106)
LPESVHLNK, VLSTSFPPK	*Kluyveromyces marxianus* protein	Trypsin and chymotrypsin (5 h)	/	(9)
VLYSTPVKMWEPGR, VITVATAGSETMR, HIGININSR	*Tinospora cordifolia* stem proteins	Papain (2 h), pepsin (2 h), Trypsin-α-chymotrypsin (3 h), and pepsin-pancreatin (2 h)	/	(107)
AHLL	Royal jelly	Simulated gastrointestinal digestion using pepsin (1 h), trypsin and chymotrypsin (2 h)	Apalbumin 2	(108)
PFPGPIPN	Pine nut	Proteinases k from *Tritirachium album*	/	(109)
LHLPLP		Proteinases k from *Tritirachium album*	/	
QKEPMIGV		Thermolysin (from *Tritirachium album*)	/	
KYIPIQ		Alcalase (from *Bacillus licheniformis*) hydrolysis	/	
LPLPLL		Trypsin (from porcine pancreas) hydrolysis	/	
SLPQNIPPL	Fermented milk	Fermentation using *Lactococcus lactis*	/	(110)
IIAEK	Bovine milk	Trypsin (pH 8.0, 6 h)	/	(111)
RLLPH	Hazelnut	Alcalase (2 h)	/	(112)
NVQR, AQMACPHL, VAPAGHAVT, PHHCDAEAI, HSDDDGQIR, TATAVV, LQR, and GTIT	cocoa	*In silico* proteolysis with simulated gastrointestinal digestion using the enzymes pepsin (2 h), pancreatin (2 h)	/	(113)
RLPSEFDLSAFLRA, RLSGQTIEVTSEYLFRH	*Pleurotus cornucopiae*	Water and methanol extraction (24 h)	/	(114)
LSMGSASLSP	*Hypsizygus marmoreus*	Water extraction at 50 °C for 12 h	//	(115)
AHEPVK	*Pleurotus cystidiosus*	Ammonium sulfate precipitation, reverse-phase HPLC and size exclusion chromatography	/	(116)
RIGLF, PSSNK	*Agaricus bisporus*		/	
WALKGYK, LLVTLKK, IISKIK, ILSKLK, LIDKVVK	*Tricholoma matsutake*	Water extraction	/	(117)
QLVP, QLDL, QDVL	*Ganoderma lucidum*	Water extraction at 4 °C and ultrafiltrated	/	(118)
Cordymin (AMAPPYGYRTPDAAQ)	*Cordyceps sinensis*	Water extraction (pH 4.5)	/	(119)
*Pleurotus eryngii* polypeptides	*Pleurotus eryngii*	PBS extraction for 4 h and filtered using 30-kDa dialysis membranes	/	(120)
Peptide mixtures	*Agaricus bisporus*	Pepsin-trypsin-α-chymotrypsin mixture for 2.5 h, then pepsin and trypsin-α-chymotrypsin mixture for 2.5 h	/	(121)
Bottom Up				
FFVAPFPEVFGK	Milk protein	Recombinant DNA technology using *Escherichia coli* at 37 °C	/	(122)
HVLPVP	Milk protein	Recombinant DNA technology using *Escherichia coli* (5 h)	/	(123)
AEEEYPDL	Spanish dry-cured ham	Synthesized	Creatine kinase	(124)
FLV	Soy	Synthetized and the purity was >96%	/	(125)
P1 (YFDEQNEQFR), P2 (GQLLIVPQ), P3 (SPFWNINAH), P4 (NINAHSVVY), and P5 (RALPIDVL)	Oat protein	Synthetized at the purity of 95% (on the basis of HPLC)	/	(126)
KSPLY	*Hericium erinaceus*	Synthesized using solid-phase method and purified by HPLC	/	(34)
IIAEK	Cow’s milk	DNA fragment coding recombination	β-lactoglobulin	(40)
KVLPVP	Milk	The gene of the dotetra-contapeptide was synthesized and expressed in *Escherichia coli* BL21 to obtain the recombinant KVLPVP	/	(123)
LFchimera peptide	Tobacco	Recombinant expression using a positively integrated and expressed LFchimera tobacco line	/	(37)
(−)-Ternatin [d-allo-Ile^1^-l-(NMe)Ala^2^-l-(NMe)Leu^3^-l-Leu^4^-l-(NMe)Ala^5^-d-(NMe)Ala^6^-(2*R*,3*R*)-3-hydroxy-Leu^7^]	*Coriolus versicolor*	Solid phase method	/	(127)

The method of using enzymatic hydrolysis of raw proteins from egg, milk, fish, fungi, and legumes is the most widely used to produce bioactive peptides. This method has many advantages such as short reaction times, high efficiency, and environmental friendliness. In this regard, more than one proteolytic enzyme (purified or crude) can be used to hydrolyze the proteins to produce peptide-containing hydrolysates. Gastrointestinal (GI) enzymes, such as trypsin, pepsin, α-chymotrypsin, and pancreatin, are widely used.^14^ Other enzymes, such as papain, neutrase, flavorzyme, and alcalase, and their combinations, have also been frequently utilized. Moreover, the simultaneous or sequential addition of the enzymes depends on their respective optimal pH, temperature, and other reaction conditions. For example, bioactive peptides of egg proteins were produced using alkaline protease with 40 mg/mL protein at pH 9.0 and 60 °C for 7 h.^15^ The bioactive peptides were produced by hydrolyzing casein using several proteases such as flavorzyme, savinase, thermolysin, trypsin, and elastase at different pressures under various enzyme-to-substrate ratios and incubation times.^16^ Codfish skin polypeptides were obtained through trypsin hydrolysis.^17^ An antioxidant peptide from pecans was produced by alcalase at 55 °C and pH 10.0 for 0–180 min.^18^

The method using microbial fermentation for the production of bioactive peptides mainly relies on the proteolytic enzymes released by the specific bacteria or yeast. The microorganisms are cultured in a medium containing protein as a substrate for growth, and the resultant peptides are generated via microbial hydrolysis of the parent proteins. This method is commonly used in the production of bioactive peptides from some dairy products, marine origins, poultry products, cereals, and legumes. The microorganisms commonly used in the fermentation process mainly include the bacteria such as *Lactococcus, Lactobacillus, Streptococcus thermophilus*, and *Bacillus subtilis*, and fungi such as *Saccharomyces* and *Aspergillus niger*.^19,20^ Usually, the microorganisms are grown to their exponential phase at the best suitable growth temperature, followed by being suspended in sterile distilled water (usually containing glucose) and then being used as a starter to inoculate sterilized protein substrates.^21,22^ For example, bovine milk peptides were obtained from the parent whey proteins through fermentation of *Lactobacillus* and *Saccharomyces*.^23^ AIPPHPYP derived from fermented fish was produced by using *Malaysian pekasam* and *Lactobacillus plantarum* at 27 °C for 15 days.^24^ A peptide with the sequence FISNHAY from porcine skeletal muscle protein was generated by fermentation of meat-borne *Lactobacillus*.^25^ The use of microbial fermentation to produce natural bioactive peptides has a competitive advantage in terms of technology and economy. However, other biological macromolecules such as proteins and polysaccharides secreted by microorganisms will affect the product purity, and the subsequent separation and purification process are relatively complicated. Therefore, it is necessary to optimize the separation process and select and breed microbial strains with high safety and a strong transformation ability in the actual production of peptides when the microbial fermentation method is the choice.

The method of *in silico* proteolysis is a technology based on bioinformatics that predicts the cleavage site of a protein sequence with a specific protease under some given conditions. Ibrahim et al. have summarized the essential structural conditions for producing active peptides with a property of inhibiting α-glucosidase, i.e., the presence of amino acids containing a hydroxyl or basic side chain at the N-terminal, proline at the penultimate C-terminal position, and alanine or methionine at the C-terminal end.^26^ The designed peptide sequences were assessed for the potential hydrolysis by the gastrointestinal digestion endopeptidase using the BIOPEP database (http://www.uwm.edu.pl/biochemia/index.php/en/biopep).^27^ Besides, with a known peptide sequence, the potential biological activities can be further predicted and identified using quantitative structure–activity relationships (QSAR) and molecular docking.^28^ The basic properties of peptides can be analyzed by several online tools, such as Expasy-Compute pI/Mw (https://web.expasy.org/compute_pi/), ProtParam (https://web.expasy.org/protparam/), and ToxinPred (http://crdd.osdd.net/raghava/toxinpred/).^29,30^ For example, milk proteins appeared to be the most frequently studied with *in silico* methodologies.^11,12,28,30,31^ The potent neuroprotective peptides from walnut protein hydrolysate were identified using *in silico* analysis in cognitive and memory impairment zebrafish.^32^ With such advantages of efficient result acquisition, less use of chemicals, and relatively high yield, this method has started to gain wide application.

The method of using chemical or enzymatic synthesis for the production of bioactive peptides is to synthesize a specific sequence peptide with its respective amino acids. As one of the chemical syntheses, solid-phase peptide synthesis (SPPS) has been widely used for producing peptides with varying lengths. The basic principle of SPPS is that the C-terminus of amino acids is fixed on the resin, followed by adding the amino acids to extend the peptide chain.^33^ This method is generally preferred when a medium-length peptide is synthesized. The peptide KSPLY from *H. erinaceus* was chemically synthesized by the solid phase method.^34^ To improve the efficiency of synthesis, a new method of using proteases to synthesize peptides was proposed by Kullman et al.^36^ They used papain and thermolysin as the catalyzing enzymes and successfully synthesized leu-enkephalin (Tyr-Gly-Gly-Phe-Leu) and met-enkephalin (Tyr-Gly-Gly-Phe-Met). Compared with that of the chemical synthesis, the most prominent advantage of enzyme-catalyzed synthesis is the specificity of amino acid selection in the reaction.^35^ However, the enzyme-catalyzed synthesis method is not widely used due to the cumbersome purification process of specific protease with high purity and high activity.

Recombinant protein expression and genetic engineering technology have begun to be employed for the production of bioactive peptides in prokaryotic and eukaryotic cells. Such methods have been used for mass production and synthesis of long chain peptides.^37^ If the target product is a short-chain peptide, it is easily degraded by proteases in microbial cells, thereby reducing the efficiency of recombinant expression and production of peptides.^38^ In the prokaryotic expression system, for example in *Escherichia coli*, two methods can be used to solve the problem of possible target peptide degradation: 1) using protease-deficient strains; 2) expressing peptides in the form of insoluble inclusion bodies or concatemers.^39^ Abundant antimicrobial and antihypertensive peptides were synthesized through genetic recombinant technology in *E. coli*.^39,40^ In the eukaryotic expression system, the yeast system is commonly used to express the peptides, especially in *Pichia pastoris*.^41−43^ Compared with the chemical synthesis, the genetic recombinant technique has a lower yield of target products, so the selection and design of expression systems are particularly important. In addition, the invalid peptide fragments or peptides secreted by the host cells may increase the difficulty to separate and purify the target peptides.

### Purification and Identification of Bioactive Peptides

After extraction and preliminary bioactivity screening, the crude peptides were obtained and subjected to further purification. Ultrafiltration membrane systems are usually used to separate peptides with the desired molecular weight by selecting the correct cutoff position.^16^ Although these systems are fast and economic, the membrane reproducibility has been reported to be poor, and the interaction between peptides and the membrane may be seen. Thereafter, one or multiple chromatographic techniques will be jointly utilized after ultrafiltration, such as liquid chromatography (LC), fast protein liquid chromatography, ultraperformance liquid chromatography, and reversed-phase-high-performance liquid chromatography.^9,16^ However, these LC methods may not be feasible for crude mixture samples containing a small number of target peptides. Therefore, multidimensional approaches, such as gel filtration chromatography (size exclusion chromatography), ion exchange chromatography, and affinity chromatography, are generally used.^128,129^ Furthermore, the appropriate combinations of the purification systems described above can shorten the purification time and enhance the recovery rate.

Generally, mass spectrometry (MS) is often used to identify and characterize peptides, attributed to the high sensitivity, accuracy, and fast processing time. The target peptides are ionized by high-energy electrons in MS, and then the molecular fragmentation passes through a mass-to-charge analyzer according to the mass-time or mass-to-charge ratio.^130^ Matrix-assisted laser desorption/ionization time-of-flight mass spectrometry (MALDI-TOF-MS), surface-enhanced laser desorption/ionization time-of-flight mass spectrometry (SELDI-TOF-MS), or liquid chromatography tandem-mass spectrometry (LC-MS/MS) are commonly used for sequence validation of target peptides.^131^ For example, several angiotensin-converting enzyme (ACE)-inhibitory peptides in Cheddar cheeses were detected by MALDI-TOF-MS.^132^

## Bioavailability of Bioactive Peptides

The digestibility, bioavailability, and metabolic fate of food-derived bioactive peptides are critical for revealing the mechanisms underlying the beneficial effects after the intake of peptides. Oral delivery remains one of the most preferred modes of bioactive peptides.^133^ However, the complex GI environment and the presence of various digestive enzymes greatly affect the bioavailability, stability, effectiveness, and potency of the bioactive peptides. In addition, studies have demonstrated that structural properties of peptides such as molecular weight, charge, hydrophobicity, and amino acid sequence have a certain degree of influence on their stabilities.^134,135^ Furthermore, other factors such as the environment pH, ionic strength, food carriers, digestive hormones, neural regulation, and gut microbiota will also affect the absorption and bioavailability of the active peptides.^133,136,137^ After GI digestion, the peptides need to cross the barriers of the intestinal epithelium, such as the impermeable epithelium, and survive after the biochemical actions of enzymes.^134^ Even though the bioavailability of food peptides is low due to gastrointestinal degradation, recent research has demonstrated that many peptides can cross the small intestine and reach the blood circulation intact.^138^ The previous research has found that bioactive peptides are transported across the GI epithelium into the blood circulation via one or more of the following pathways: 1) peptide transporter 1 (PepT1)-mediated transport, 2) transcytosis via vesicles, 3) a paracellular route via tight junctions, and 4) passive transcellular diffusion.^136,139^

Cell line models (Caco-2 cells), excised tissues mounted on a chamber (animal, ex vivo), and intestinal perfused loops (animal, in situ) have been used to study the transportation and absorption of food peptides to simulate the human intestinal membrane.^140^ Normally, the permeabilities of the bioactive peptides are low, approximately less than 1%.^141^ The *in vitro* studies using cell models do not fully consider the digestive and metabolic processes, resulting in inconsistent doses. To validate the *in vitro* results, animal and human studies should be conducted. It has been shown that some bioactive peptides could survive from gastrointestinal degradation and reach blood circulation and their target organs intact.^138,142^ Normally, dipeptides and tripeptides are absorbed intact by epithelial cells.^143^ For example, the dipeptide VY has been detected in the plasma, heart, liver, and kidney of rats with an elimination half-life of about 3.1 h.^144^ On the contrary, most peptides with more than three amino acids are hydrolyzed by peptidases after transporting to the GI tract and intestinal epithelium. As summarized by a recent review, the maximum concentrations (*C*_max_) of most peptides in the plasma of humans or animals are approximately in the micromolar range, which is far below that used for *in vitro* assays (approximately 1–100 μM).^14^ Interestingly, many *in vivo* studies have shown that oral intake of bioactive peptides possesses benefits and bioactivity, even though they have a low bioavailability. Regarding oligopeptides (tetrapeptides or longer peptides), their stabilities, kinetics, and bioavailability remain largely unknown. Besides, it has been proposed that some food-derived peptides might be metabolized into other active metabolites in the body with different biological activities.^6^ Hence, the dose, stability, bioavailability, and metabolism should be taken into consideration in assessing the benefits of peptides.

## Bioactive Peptides As Enzyme Inhibitors

*In vitro* enzyme testing has been commonly used for activity-guided fractionation and to isolate and identify active peptides. A summary of *in vitro* studies regarding the enzymatic test is shown in Table 2. As an effective prophylactic treatment of hyperglycemia and diabetes, inhibition of α-amylase and α-glucosidase can reduce the hydrolysis of carbohydrates and consequently decrease the absorption of glucose into the circulation.^145^ Bioactive peptides from egg yolk protein,^44^ egg white ovalbumin,^45^*Spirulina platensis*,^146^ germinated soybean,^96^ and amaranth protein^106^ were strong inhibitors on α-glucosidase, while bioactive peptides from chickpea,^102^*Spirulina platensis*,^146^ germinated soybean,^96^ and oat protein^126,151^ were potent in inhibiting α-amylase. Inhibition of DPP-IV may exhibit antihyperglycemic activity by blocking the rapid breakdown of incretin. Such inhibition effects were observed in peptides obtained from proteins of milk,^147^ goat (*Capra hircus*) milk,^147,148^ meat,^149^ yogurt,^54^*Atlantic salmon*,^75^ egg yolk,^44^*Spirulina platensis*, chickpea,^102^ amaranth,^106^*Sardine pilchardus*,^77^ whey, *Antarctic krill*,^150^ germinated soybean,^96^ soy, and lupin.^101^ Besides, ACE is a well-known enzyme that can convert inactive angiotension I (Ang-I) to active Ang-II. In this regard, inhibition of ACE could lead to the control of blood pressure in T2D. Bioactive peptides from proteins of egg yolk and chickpea demonstrated an excellent ACE-inhibition activity.^102^ As lipase catalyzes the decomposition of triacyclglycerols (TG) to glycerol and fatty acids, bioactive peptides isolated from proteins of amaranth and oat could inhibit lipase in the intestine, reduce fat absorption, and have antiobese activity.^106,151^ These facts indicate that bioactive peptides have the potential to display multiple physiological functions.

**Table 2:** *In Vitro* Inhibiting Activity of Bioactive Peptides on Relevant Enzymes Related to Obesity and Diabetes^a^

peptides	sources	main findings/mechanism and effect	reference
YIEAVNKVSPRAGQF, YINQMPQKSRE, VTGRFAGHPAAQ	Egg yolk protein	↓ACE, α-glucosidase, and DPP-IV activities	(44)
		↑DPPH free radical scavenging	
RVPSL	Egg white ovalbumin	↓α-glucosidase with an IC_50_ value at 23.07 μmol/L	(45)
GVPMPNK, RNPFVFAPTLLTVAAR, LRSELAAWSR	*Spirulina platensis*	↓DPP-IV, α-glucosidase, and α-amylase activities	(146)
YVDGSGTPLT, PHPATSGGGL, GKAAPGSGGGTKA	Chickpea protein	↓ACE, DPP-IV, and α-amylase activities	(102)
FPFPPTLGY, FPFPR	Amaranth protein	↓DPP-IV and α-glucosidase activities	(106)
NNDDRDS, LSSTEAQQS, NAENNQRN, QQQQQGGSQSQ, EEPQQPQQ, IKSQSES	Germinated soybean	↓DPP-IV, α-amylase and α-glucosidase	(96)
RLLPH	Hazelnut (*Corylus heterophylla* Fisch) protein	↓Pancreatic lipase activity	(112)
YP, LP, IPI, VPL, IPA, IPAVF	Milk protein	↓DPP-IV activity	(147)
INNQFLPYPY, MHQPPQPL	Goat (*Capra hircus*) milk	↓DPP-IV activity	(58)
PPL	Meat protein	↓DPP-IV activity	(149)
PPFLQPEVM, FVAPFPEVF	Yogurt	↓DPP-IV activity	(54)
GPAV	Atlantic salmon	↓DPP-IV activity	(75)
LKPTPEGDL, LPYPY, IPIQY, IPI, WR	Milk protein	↓DPP-IV activity	(148)
NAPNPR, YACSVR	*Sardine pilchardus* protein	↓DPP-IV activity	(77)
LDQWLCEKL	Trypsin-hydrolyzed α-lactalbumin-rich whey proteins	↓DPP-IV activity	(59)
AP, IPA	Antarctic krill (*Euphausia superba*) protein	↓DPP-IV activity	(150)
YFDEQNEQFR, GQLLIVPQ, SPFWNINAH, NINAHSVVY, RALPIDVL	Oat protein	SPFWNINAH: the best lipase inhibitor (IC50 85.4 ± 3 μM);	(151)
		YFDEQNEQFR: the most potent inhibitor of α-amylase (IC50 37.5 ± 1.1 μM)	
IAVPTGVA, YVVNPDNDEN, YVVNPDNNEN	Soy	Soy peptide IAVPTGVA ↓ DPP-IV activity by 46%;	(89)
		YVVNPDNDEN and YVVNPDNNEN were inactive against DPP-IV.	
LTFPGSAED, LILPKHSDAD, GQEQSHQDEGVIVR	Lupin	Lupin peptide LTFPGSAED ↓ DPP-IV activity by 46%	
		LILPKHSDAD and GQEQSHQDEGVIVR were inactive against DPP-IV.	

a Abbreviations: ACE, angiotensin converting enzyme; DPP-IV, dipeptidyl peptidase IV; DPPH, 1,1-diphenyl-2-picrylhydrazyl; IC value, the peptide concentration that inhibits 50% activity.

## Effects of Bioactive Peptides in *in Vitro* Studies

Both hypertrophic adipocytes and adipose macrophages in obesity can cause inflammation and enhance insulin resistance by releasing inflammatory cytokines, resulting in the occurrence of metabolic diseases.^152,153^ Several signaling pathways, such as AMP-activating protein kinase (AMPK)/acetyl-CoA carboxylase (ACC), phosphatidylinositide 3-kinase (PI3K)/protein kinase B (Akt)/glycogen synthase kinase 3b (GSK3b), and peroxisome proliferator-activated receptors (PPAR) regulation, have been shown to be associated with adipocyte differentiation, lipid metabolism, and/or the insulin signaling pathway.^154,155^ Commonly, PepT1 and PepT2 on the membrane modulate the cellular uptake of di- or tripeptides. Kwak et al. found that PepT2 played significant roles in promoting the uptake of soy peptide FLV in adipocytes.^125^ To further evaluate the potential antidiabetes and antiobese effects of peptides, numerous *in vitro* studies on cell culture have been conducted in macrophage RAW 264.7 cells, human adipocytes such as the classical 3T3-L1 adipocytes and preadipocyte cell line 3T3-F442A, muscle cells such as C2C12 myotubes and rat-derived L6 myoblasts, as well as insulin resistance hepatocytes (HepG2 cells). The amino acid sequence, source, doses, and main effects of relevant bioactive peptides are summarized in Table 3.

**Table 3:** *In Vitro* Studies of Peptides and Their Effects Related to Diabetes and Obesity^a^

peptides	source	subject	treatment details	main findings/mechanism and effect	reference
VPP	Milk	Coculture of 3T3-L1 adipocytes and RAW264 macrophages	1 mM VPP added into cocultures for 8 days	↓TNF-α expression induced by ACE overexpression in 3T3-L1 adipocytes	(48)
				↓TNF-α and IL-1β expression in RAW264 macrophage and 3T3-ACE adipocyte cocultures, but not in RAW264-3T3-GFP adipocyte cocultures.	
LPDEVSG, DDNKVED, GVDTKSD, IESGSVEQA, GGLVVT	Pepsin treated egg white hydrolysate	LPS treated mouse macrophage cells RAW 264.7	0, 12.5, 25, 50, 100, 200 μg/mL	↓ACE	(46)
FRADHPPL, FSL, SALAM, YQIGL, RADHPFL, IVF, YAEERYPIL, YRGGLEPINF, RDILNQ, ESIINF	Peptidase treated egg white hydrolysate			↓ ROS, CHOL, and IL-6	
FLV	Soy	Marine macrophages cells RAW 264.7 and 3T3-L1 adipocytes	0.1 or 1 μM	↓Release of inflammatory cytokines (TNFα, MCP-1, and IL-6) from both TNFα-stimulated adipocytes and cocultured adipocytes/macrophages, regulated by ↓JNK, IKK, and IκB	(125)
				↑Insulin responsiveness and glucose uptak	
(−)-Ternatin [d-allo-Ile^1^-l-(NMe)Ala^2^-l-(NMe)Leu^3^-l-Leu^4^-l-(NMe)Ala^5^-d-(NMe)Ala^6^-(2R,3R)-3-hydroxy-Leu^7^]	*Coriolus versicolor*	3T3-L1 adipocytes	1 μg/mL	↓Fat accumulation against 3T3-L1 murine adipocytes	(127)
IQN	Black soybean (*Rhynchosia volubilis* Lour.) hydrolysate	3T3-L1 adipocyte	NA	↓Differentiation of the 3T3-L1 preadipocyte	(165)
GAGAGY	Fibroin	Insulin-resistant 3T3-L1 adipocytes	1 mg/mL	↑Phosphorylation of Akt and GLUT4 translocation;	(166)
				↑PI3K signaling pathway	
				↓Development of insulin resistance	
EITPEKNPQLR, RKQEEDEDEEQQRE	Purified soybean β-conglycinin	3T3-L1 preadipocytes	0, 12, 25, 50 μM	↓FAS activity and *de novo* fatty acid synthesis in adipocytes	(178)
GAGVGY	Fibroin	3T3-L1 adipoctyes	0, 0.1, 0.5, 1, 2 mg/mL	↑Basal and insulin-stimulated glucose uptake through ↑GLUT1 mRNA and GLUT4 translocation;	(171)
				↓Expression of SREBP1c, PPARγ, FAS, corroborated with ↓lipid accumulation	
				↓mRNA expressions of PPARγ, C/EBPα, SCD1, and FAS	
				↑AMPK phosphorylation and adiponectin secretion	
EQRPR	Rice bran	Undifferentiated human preadipocytes	NA	Showed 70% adipocyte viability and a protective role against obesity	(218)
/	Silk and silkworm pupa	3T3-L1 preadipocytes	1, 10, or 50 μg/mL	↓Adipogenesis	(174)
				↓Expression of leptin and Acrp30 mRNA	
				↑Production of adipogenesis-related proteins PPARγ and Acrp30	
ILL, LLL, VHVV	Flavourzyme-soy protein isolate	3T3-L1 preadipocytes	400 ppm (mg/kg)	Showed notable lipolysis-stimulating activity by exhibiting better glycerol release	(90)
DIVDKIEI	Tuna	3T3-L1 adipocytes	0, 500, 1000 ng/mL	↓Adipocyte differentiation	(172)
				↓mRNA levels of C/EBP-α and PPARγ;	
				↓Lipid components and adipogenesis.	
DIVDKIEI	Tuna	3T3-L1 mouse preadipocytes	0, 125, 250, 500 and 1,000 ng/mL	↓C/EBP mRNA expression	(219)
				↑Wnt-10b mRNA expression	
				↓Glucose uptake and TG levels	
				↓Adiponectin and high-molecular weight adiponectin levels	
Subcritical water-hydrolyzed fish collagen peptide	Fish collagen	Mouse 3T3-L1 preadipocytes and human hepatoblastoma-derived HepG2 cells	0, 0.2, 0.5, 1, 2, 5 mg/mL	↓Fat accumulation during the differentiation of 3T3-L1 preadipocytes	(173)
				↓Expression of C/EBP-α, PPARγ, and αP2 genes	
				↓The palmitate-induced accumulation of lipid vacuoles in hepatocytes	
NALKCCHSCPA, LNNPSVCDCDC, MMKAAR, NPVWKRK, CANPHELPNK	*Spirulina platensis*	Mouse 3T3-L1 preadipocytes and liver cells (L-O2)	0, 200, 400, and 600 μg/mL	↓3T3-L1 preadipocytes proliferation	(88)
				↓Accumulation of TG	
IPP, VPP	Fermented milk	3T3-F442A preadipocytes	50 μM	Showed insulin sensitizing effect in adipocytes through ↑Akt and ERK1/2 phosphorylation;	(167)
				↓NF-kB under TNF stimulation	
				↑GLUT4 in adipocytes and restored glucose uptake in TNF-treated adipocytes.	
VFVRN	Chickpea	3T3-L1 preadipocytes	1, 2, 3 mmol/L	↑3T3-L1 preadipocyte apoptosis via modulating BaX, Caspase-3 and Bcl-2 expressions	(220)
RLLPH	Hazelnut (*Corylus heterophylla* Fisch)	3T3-L1 preadipocytes	0, 20, 40, and 80 mM	↓mRNA expressions of PPARδ, C/EBP-α, αP2, SREBP-1c, FAS, ACC1, HMGCR to attenuate adipogenesis	(112)
				↑Phosphorylated AMPK levels and its substrate ACC	
Viscothionin	Mistletoe (*Viscum album* var. *coloratum* Ohwi)	3T3-L1 preadipocytes	5 μM	↓Differentiation of adipocyte cells and alleviated accumulation of intracellular lipids via activating AMPK, by down-regulating phosphorylation in AKT and GSK3β;	(179)
				↓Levels of SREBP-1 and FAS	
A whey peptide mixture	Pepsin-pancreatin digestion hydrolysis from whey protein	3T3-L1 preadipocytes and C2C12 myotubes	2.5 mg/mL	↑3T3-L1 adipocyte differentiation and TG accumulation with ↑PPARγ	(160)
				↑Lipolysis and fat oxidation in adipocytes with ↑PPARδ	
				↓Palmitate-induced inflammation, insulin resistance, and diacylglycerol accumulation with ↑sequestration of fatty acids in C2C12 myotubes	
AAWKLLKALAKAAL-NH2 (αAL14)	Gill of the abalone (*Haliotis discus discus*)	3T3-L1 preadipocytes	0, 5, 10, 15, and 20 μM	↓Accumulation of lipid droplets during adipocyte differentiation	(180)
				↓Expression of PPARγ, C/EBPα, and SREBP1	
				↓PI3K/Akt signaling and its downstream factors such as mTOR, GSK-3β, and Fox	
KILDK	Bovine α-lactalbumin	3T3-L1 adipocytes	NA	↓Insulin resistance	(169)
				↓JNK phosphorylation (Thr183/Tyr185)	
				↓Pro-inflammatory genes through blocking NF-κB signaling	
Peptide fractions	Soybean	Muscle L6 cells	1 ng/mL, 1 μg/mL	↑Glucose uptake, correlated to the activation of the AMPK enzyme	(213)
Peptide fractions	Common bean (*Phaseolus vulgaris* L.)	Rat insulinoma INS-1E cells, adipocytes 3T3-L1	1, 10, and 100 μg/mL	↑Glucose-stimulated insulin secretion, ↓expression of DPP-IV, RAGE, and oxygen species from rat insulinoma INS-1E cells	(221)
				↓Lipid accumulation, ↑glucose uptake via Akt modulation, enhancing insulin signaling, and reducing PTEN activation in mature adipocytes 3T3-L1	
IRW, IQW, LPK	Egg white	Rat-derived L6 myoblasts	100 μm of IRW, IQW, or LKP in serum free DMEM	IRW: ↓glucose uptake induced by Ang-II, normalized serine phosphorylation of IRS and GLUT4 expression and ↑p-AKT	(47)
				IRW: ↓AT1R; ↓ ROS, and NADPH activity	
				IQW and LPK peptides had antioxidant activities	
Viscothionin	Mistletoe (*Viscum album* var. *coloratum* Ohwi)	Rat insulinoma RINm5F cells and C2C12 myoblast cells	0, 100, 500, and 1000 μg/mL	↑Insulin secretion from RINm5F cells	(212)
				↑Secretion of insulin and C-peptide by RINm5F cells and ↑expression of GLUT-4, IRS-1, and AKT in differentiated C2C12 cells	
7S-peptides	Highly purified soybean β-conglycinin (7S)	HepG2 cells	0, 3, 6 mg/mL	↓Secretion of apolipoprotein B-100	(205)
				↓Incorporation of 3H-glycerol and 14C-acetate into TG	
				↓Production of cholesterol esters and intracellular cholesterol levels	
				↑Genes expressions associated with fatty acids β-oxidation and cholesterol synthesis	
LA, VL, ST	Soy protein isolate	HepG2 cells	0, 1, 10 mg/mL	↓TG synthesis and apoB secretion	(206)
IAVPGEVA, IAVPTGVA, LPYP	Soy	HepG2 cells	500 μM	Regulate glucose metabolism	(91)
				↑Glucose uptake via activating GLUT1 and GLUT4 by stimulation of AKT and AMPK pathways	
IPPKKNQDKTE	Casein glycomacropeptide	High glucose-induced insulin resistant (IR) hepatic HepG2 cells	0, 125, 250, and 500 μM	↑Phosphorylation of Akt and GSK3β	(192)
				↓Expressions of p-GS, G6 Pase, PEPCK	
				↓IRS-1 Ser307 phosphorylation	
				↑Phosphorylation of AMPK	
IPPKKNQDKTE	Casein glycomacropeptide	High glucose-induced IR hepatic HepG2 cells	0, 125, 250, and 500 μM	↑Insulin-stimulated glucose uptake	(192)
				↓High glucose-induced ROS production	
				↓Activation of MAPK signaling	
				↑Nrf2 nucleus translocation and HO-1 expression	
LPLLR	Walnut (*Juglans mandshurica* Maxim.)	High glucose-induced IR hepatic HepG2 cells	100 and 200 μM	↓α-glucosidase and α-amylase	(193)
				↑Phosphorylation levels of IRS-1, PI3K, Akt, and GSK3β	
				↑Thr172 phosphorylation of AMPKα	
				↓Expression of TORC2, CREB, and PGC-1α	
				↑Expression levels of GS and GLUT4	
				↓Expression levels of G6 Pase and PEPCK	
LVRL, LRYL, and VLLALVLLR	Walnut (*Juglans mandshurica* Maxim.)	High glucose-induced IR and oxidative stress in hepatic HepG2 cells	100 and 200 μM	↑Glucose consumption, glucose uptake, and GLUT4 translocation	(222)
				↑Phosphorylation of IRS-1, PI3K, and Akt	
				↑Activities of GSH-Px, CAT, SOD, Nrf2, and HO-1	
				↓ROS overproduction and the phosphorylation of ERK, JNK, and p38	
GPPGPA	Collagen hydrolysates of Chinese giant salamander skin	Insulin resistant HepG2 cells	NA	↓Insulin resistance	(202)
				↑Activity of SOD	
				↓Content of malondialdehyde (MDA)	

a Abbreviations: ACC1, acetyl-CoA carboxylase 1; ACE, angiotensin converting enzyme; AKT, protein kinase B; AMPK, activated protein kinase; Ap2, adipocyte fatty acid-binding protein; AT1R, angiotensin II type 1 receptor; AT2R, angiotensin II type 2 receptor; C/EBP-α, CCAAT/enhancer binding protein alpha; CCL5, CC chemokine ligand 5; CHOL, Cholesterol; CREB, cAMP-response element binding protein; DPPH, 1,1-diphenyl-2-picrylhydrazyl; DPP-IV, dipeptidyl peptidase IV; ERK1/2, extracellular signal regulated kinase 1/2; FAS, fatty acid synthase; GLUT1, glucose transporter 1;GLUT4, glucose transporter 4; GSK3β, glycogen synthase kinase 3β; HMGCR, 3-hydroxy-3-methylglutaryl-CoA reductase; IL-1β, interleukin 1β; IL-6, interleukin 6; IκB kinase; IRS, insulin receptor; IRS-1, insulin receptor 1; IKK, JNK, c-Jun N-terminal kinase; MCP-1, monocyte chemoattractant protein-1; NA, not available; NADPH, nicotinamide adenine dinucleotide phosphate; PPARγ, peroxisome proliferator associated receptor gamma; PTEN, phosphatase and tensin homologue deleted on chromosome ten; ROS, reactive oxygen species; SCD1, stearoyl CoA desaturase 1; SREBP1c, sterol regulatory element binding protein 1c; TG, triglyceride; TNF-α, tumor necrosis factor alpha.

### Inhibition of Inflammation in Macrophages and Adipocytes

A low-grade systemic chronic inflammatory response is associated with obesity by activating immune cells and releasing inflammatory cytokines and adipokines.^156^ The food-derived peptides were found to have anti-inflammatory activities in macrophage RAW 264.7 cells and adipocytes; for example, a peptide VPP derived from milk decreased the expression of tumor necrosis factor-α (TNF-α) and interleukin-1β (IL-1β) in RAW264 macrophage and 3T3-ACE adipocyte cocultures with a concentration of 1 mM;^48,157^ peptides obtained after pepsin hydrolysis of egg white could decrease the activity of ACE, while peptides produced from peptidase hydrolysis of egg white inhibited the release of proinflammatory cytokines, especially IL-6 with curbing reactive oxygen species (ROS) and cholesterol.^46^ It was worth noting that VPP had a low permeability, and only 2% intact VPP was transported via paracellular passive diffusion through *in vitro* Caco-2, *ex vivo* Ussing chamber, and *in situ* intestinal perfusion experiments.^158^ Furthermore, a soy tripeptide with the sequence Phe-Leu-Val inhibited the secretion of TNF-α, monocyte chemoattractant protein-1 (MCP-1), and interleukin-6 (IL-6) in adipocytes, and cocultured adipocytes/macrophages. Such an anti-inflammatory effect was regulated via inactivation of the C-Jun N-terminal kinase (JNK)/IκB kinase (IKK) signaling pathway and downregulation of nuclear factor of kappa light polypeptide gene enhancer in B-cells inhibitor, alpha (IκBα) in adipocytes.^125^

### Inhibition of Adipogenesis and Stimulation of Lipolysis in Adipocytes

**Figure 2:**
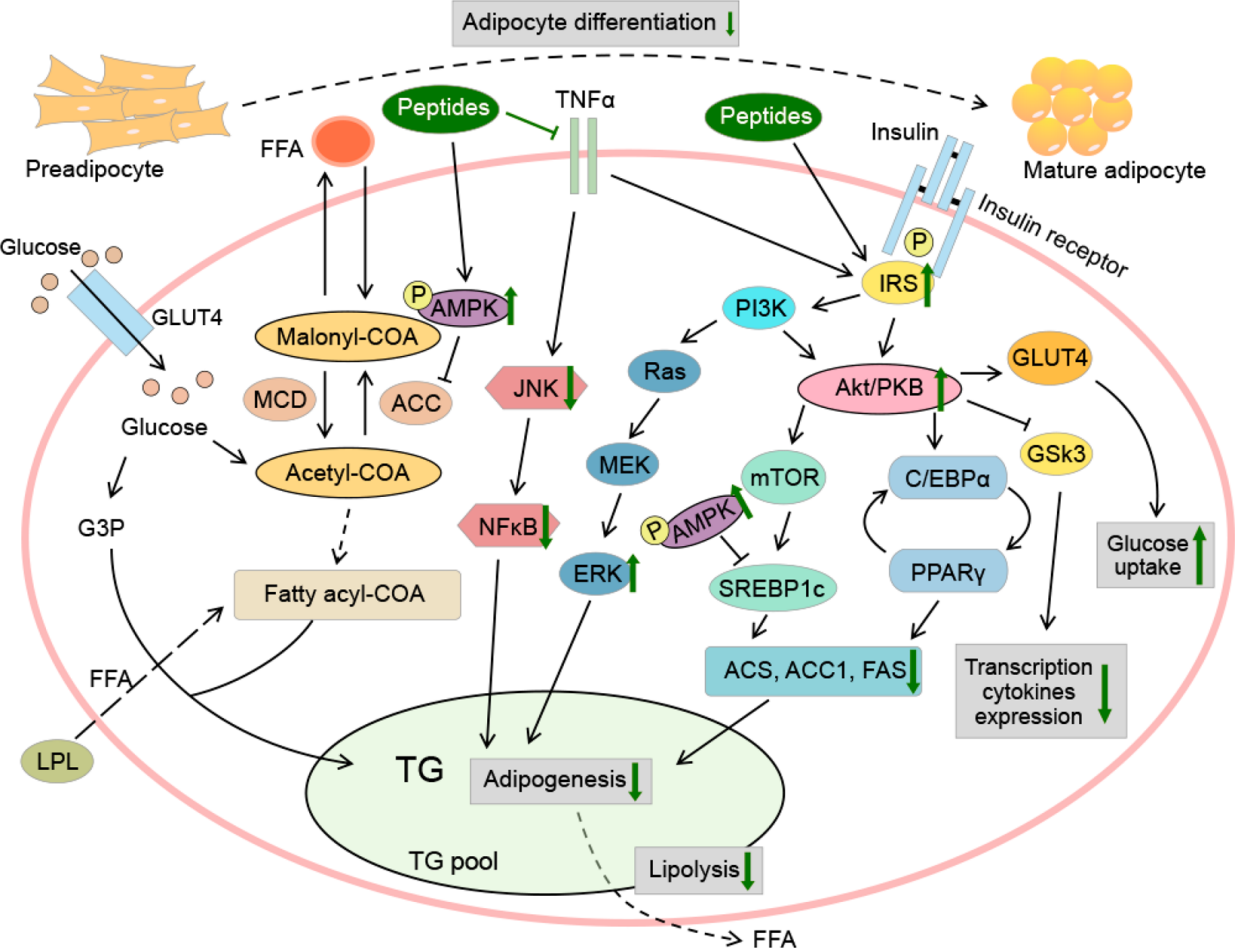
Mechanism of food-derived bioactive peptides inhibiting adipocyte differentiation and adipogenesis, and stimulating lipolysis in adipocytes. The insulin signaling pathway, PPAR regulation, and AMPK pathway are the main mechanisms. Peptides could activate the insulin/IRS/PI3K/Akt/PKB signaling pathways, subsequently promoting glucose uptake, and alleviating cytokine expression, adipogenesis, and lipolysis. Peptides could downregulate the expression levels of C/EBP-α and PPARγ to inhibit adipocyte differentiation and adipogenesis. Activation of AMPK by peptides could inhibit ACC and SREBP1c to suppress the fatty acid synthesis and adipogenesis. ACC, acetyl-CoA carboxylase; AKt, protein kinase B; AMPK, activated protein kinase; C/EBP-α, CCAAT/enhancer binding protein alpha; ERK, extracellular signal regulated kinase; FAS, fatty acid synthase; FFA, free fatty acids; G3P, glycerol-3-phosphate; GLUT4, glucose transporter4; GS, glycogen synthase; GSK3, glycogen synthase kinase 3; IRS, insulin receptor substrate; JNK, c-Jun N-terminal kinase; LPL, lipoprotein lipase; MCD, malonyl-CoA decarboxylase; MEK, Ras/Raf/mitogen-activated protein kinase; NF-κB, nuclear factor kappa light chain enhancer of activated B cells; PI3K, phosphatidylinositide 3-kinase; PKB, protein kinase B; SREBP1c, sterol regulatory element binding protein 1c; PPARγ, peroxisome proliferator associated receptor gamma; TG, triglyceride; TNF-α, tumor necrosis factor alpha; mTOR, mammalian target of rapamycin.

The 3T3-L1 cell line is a well-established and standard model to investigate adipogenesis. The 3T3-L1 preadipocytes can achieve a complete differentiation into mature adipocytes through the accumulation of lipid droplets, in which excess energy is stored in the form of TG by simple esterification from dietary fatty acids.^159^ The expansion of white adipose tissue involves adipogenesis (differentiation of preadipocytes into adipocytes), lipogenesis (conversion of acetyl-CoA to fatty acids), and TG synthesis (conversion of fatty acids into TG). Glycerol release is a marker of lipolysis. It was found that peptides ILL, LLL, and VHVV isolated from flavorzyme-soy protein showed significant lipolysis-stimulating activities through exhibiting a better glycerol release. Their lipolysis-stimulating activities were not influenced after pepsin and pancreatin digestion.^90^ A peptide mixture from whey proteins promoted lipolysis and mitochondrial fat oxidation with an increase in peroxisome proliferator-activated receptor delta (PPARδ) in adipocytes.^160^ The lipogenesis involves multiple signaling pathways, and the antiadipogenic properties of the food-derived bioactive peptides are summed up in Figure 2. The associated mechanisms are discussed below.

Modulation of the insulin signaling pathway is one of the mechanisms by which bioactive peptides regulate adipogenesis and lipolysis. Binding of insulin to its receptor (IR) induces tyrosine phosphorylation of insulin receptor substrate (IRS) and subsequently activates PI3K and PKB/Akt signaling pathways.^161,162^ The activated pathway facilitates the transport of glucose by stimulating glucose transporter 4 (GLUT4) and activates the mammalian target of rapamycin (mTORC1), a protein complex that functions as an activator of sterol regulatory element-binding protein (SREBP1c) for the induction of lipogenic pathways.^163^ PI3K kinases can be activated and subsequently activate p38 mitogen-activated protein kinases (MAPK) to promote adipocyte differentiation and synthesis. A heptapeptide (−)-Ternatin from *Coriolus versicolor*, a peptide IQN from black soybean, and peptides from *Spirulina platensis* significantly decreased the fat accumulation and differentiation of 3T3-L1 preadipocytes.^127,164,165^ Peptides GAGAGY from fibroin blocked the progress of insulin resistance by enhancing the phosphorylation of Akt and GLUT4 translocation and promoting the PI3K signaling pathway.^166^ Peptide fractions derived from common bean (*Phaseolus vulgaris* L.) promoted glucose-stimulated insulin secretion and glucose uptake via Akt modulation.^47^ Except for the intracellular signaling cascades in the PI3K, insulin signaling also activates extracellular signal regulated kinase (ERK) pathways. Tripeptides IPP and VPP exerted an insulin-sensitizing effect through enhancing Akt and ERK1/2 phosphorylation and inhibited the inflammatory mediator NF-κB in TNF-treated adipocytes.^167^ Besides, some pro-inflammatory factors, such as IL-6, TNFα, and MCP-1, affect insulin signaling in a way independent of IRS1, i.e., activating the inflammatory signaling pathway such as JNK and nuclear factor kappa light chain enhancer of activated B cells (NF-κB) in adipose tissue.^168^ The soy peptide FLV was found to enhance insulin responsiveness and glucose uptake and decreased the release of TNF-α, MCP-1, and IL-6 via inactivation of JNK and IKK and downregulation of IκBα in TNF-α treated 3T3-L1 adipocytes.^125^ Bovine α-lactalbumin-derived peptide KILDK attenuated insulin resistance via inhibiting JNK phosphorylation (Thr183/Tyr185) and downregulating pro-inflammatory cytokines by blocking NF-κB signaling.^169^

Modulation of PPAR activity is one of the mechanisms associated with the benefits of bioactive peptides. Insulin also promotes adipogenic activity by upregulating both PPARγ and CCAAT/enhancer-binding protein α (C/EBP-α) in adipocytes. The Akt/PkB signaling pathway activates the mTORC1 and subsequently activates SREBP1c, a transcription factor for controlling the expression of PPARγ to induce lipogenesis.^163^ PPARγ and C/EBP-α play critical roles in adipocyte differentiation and adipogenic regulation through modulating lipid metabolizing enzymes such as ATP-citrate lyase (ACL), ACC1, lipoprotein lipase (LPL), and fatty acid synthase (FAS).^170^ The fibroin-originated peptide GAGVGY significantly reduced the mRNA expression of adipogenic genes, including PPARγ, C/EBP-α, stearoyl-CoA desaturase-1 (SCD1), and FAS with a reduction of lipid accumulation.^171^ The peptide DIVDKIEI from tuna protein showed the inhibition of adipocyte differentiation and adipogenesis via decreasing the expression levels of C/EBP-α and PPARγ in 3T3-L1 cells.^172^ The subcritical water-hydrolyzed fish collagen peptide suppressed lipid accumulation during the differentiation of 3T3-L1 preadipocytes by downregulating the expression of C/EBP-α, PPARγ, and fatty acid-binding protein 4 (FABP4 or αP2) genes.^173^ Peptide RLLPH from hazelnut (*Corylus heterophylla* Fisch) significantly decreased the mRNA expressions of PPARδ, C/EBP-α, αP2, SREBP1c, FAS, ACC1, 3-hydroxy-3-methylglutaryl-coenzyme A reductase (HMG-CoA-R) to attenuate adipogenesis.^112^ However, the effects of peptides on PPARγ activation varied in different studies. Conversely, the silk and silkworm pupa peptides showed an inhibition on adipogenesis, but it increased the production of PPARγ.^174^ The whey peptides facilitated the differentiation and TG accumulation in 3T3-L1 adipocytes, accompanied by increasing protein levels of PPARγ and PPARδ.^160^

AMPK is an energy sensor and can be activated and phosphorylated under the conditions of energy stress (fasting, prolonged exercise, etc.) or in response to changes in hormones (glucagon, ghrelin, etc.).^175^ Activated AMPK exerts multiple benefits in modulating insulin sensitivity and energy homeostasis. On the one hand, it inhibits ACC activity, resulting in a reduction in malonyl-CoA levels and thus inhibiting fatty acid synthesis (*de novo* lipogenesis). ACC catalyzes the synthesis of malonyl-CoA from acetyl-CoA, and malonyl-CoA decarboxylase (MCD) catalyzes the conversion of malonyl-CoA back to acetyl-CoA.^176^ On the other hand, active AMPK regulates the expression of FAS through down-regulation of SREBP1c to participate in the control of lipogenesis.^177^ Food-derived peptides, such as GAGVGY and RLLPH, have been proven to activate the phosphorylation of AMPK and its substrate ACC in 3T3-L1 adipocytes.^112,171^ Besides, EITPEKNPQLR and RKQEEDEDEEQQRE from purified soybean β-conglycinin inhibited FAS activity and *de novo* fatty acid synthesis in adipocytes.^178^

The effects of bioactive peptides on inflammation, lipid metabolism, and insulin resistance in cell cultures are normally mediated by multiple signaling pathways. Viscothionin, originated from mistletoe (*Viscum album* var. *coloratum Ohwi*) proteins, inhibited the differentiation of adipocyte cells and prevented accumulation of intracellular lipids via activating AMPK and down-regulating phosphorylation in AKT and GSK3β, accompanied by a decrease of SREBP-1 and FAS levels.^179^ A peptide named αAL14 (AAWKLLKALAKAAL-NH2) derived from the gill of abalone decreased the accumulation of lipid droplets and the expression of PPARγ, C/EBP-α, and SREBP1 during adipocyte differentiation and blocked PI3K/Akt signaling and its downstream factors such as mTOR, GSK-3β, and Fox transcription factors.^180^ Except for the above-mentioned signaling pathways, the glucose metabolic pathway is also an important mediator, which provides the required substances for TG synthesis. Glycerol-3-phosphate (G3P) is synthesized from dihydroxyacetone phosphate (a critical metabolite in the glycolysis pathway) by the enzyme G3P dehydrogenase.^181^ The fatty acyl-CoA derived from free fatty acids are re-esterified to form TG using G3P as a starting material. Glucose metabolism is an indirect pathway for clarifying the antiadipogenesis mechanism of peptides, which need to be further investigated.

### Modulation of Glucose and Lipid Metabolism in Hepatocytes

The HepG2 cell is a suitable cell line for assessing glucose uptake and metabolism because it has a blunted response to insulin and a high expression in glucose transporter 1 (GLUT1).^182^ The insulin-resistant states of hepatocytes are always related to changes of glucose metabolism, including glucose uptake, glycogen synthesis, and gluconeogenesis. Glycogen synthase (GS) is the key enzyme that controls the rate of glycogen synthesis and is modulated by GSK3β. In this connection, GSK3β can inactivate GS through phosphorylation, and thus it negatively modulates the glycogen synthesis.^183^ Moreover, two key gluconeogenic enzymes, phosphoenolpyruvate carboxykinase (PEPCK) and glucose-6-phosphatase (G6 Pase), are associated with hepatic insulin resistance, and the overexpression of these enzymes will promote the elevated gluconeogenesis.^184^

**Figure 3:**
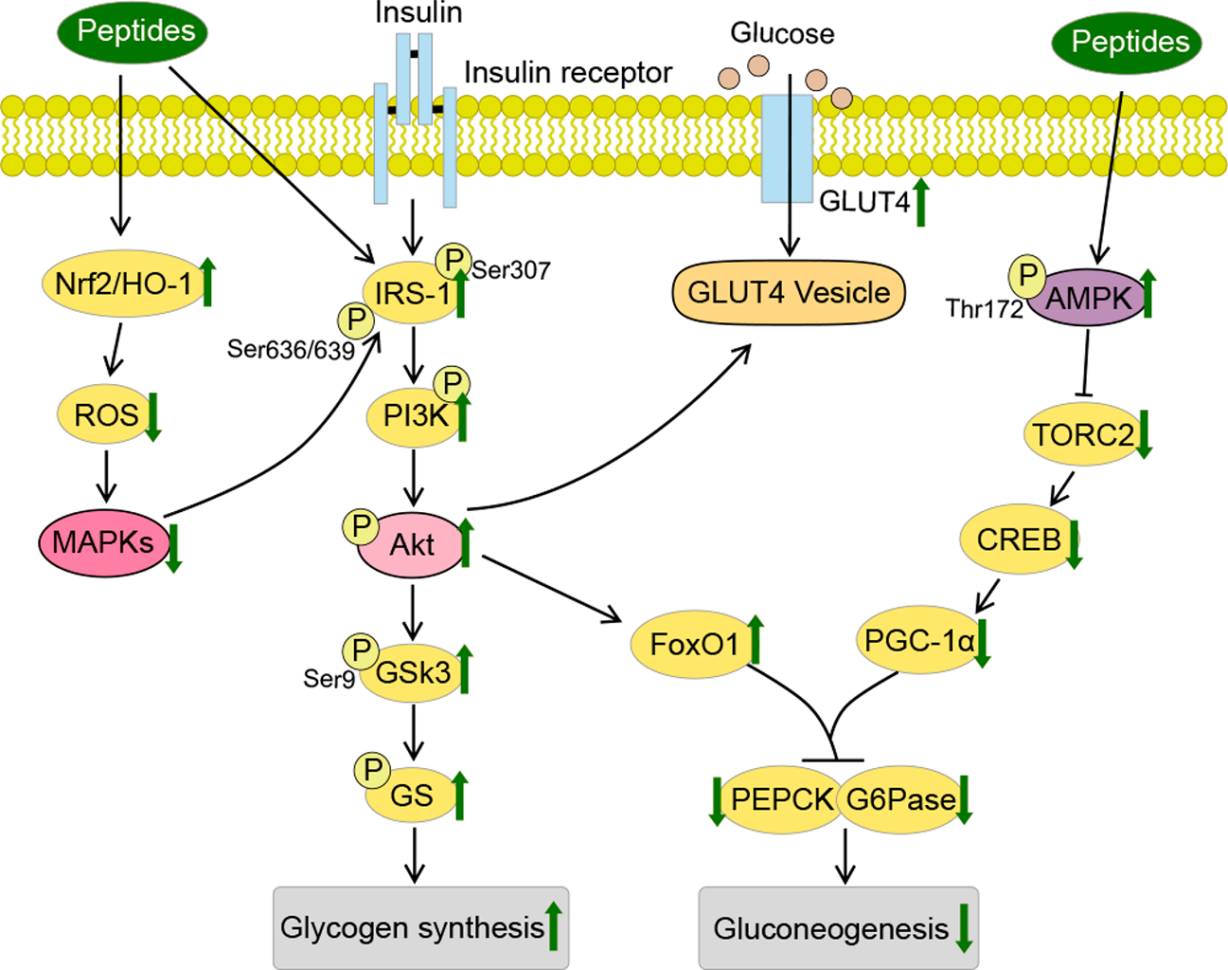
Potential action mechanism of peptides on glycogen synthesis and gluconeogenesis in hepatocytes. Bioactive peptides could promote glycogen synthesis and glucose uptake, and reduce gluconeogenesis through activating the IRS-1/PI3K/Akt and AMPK signal pathways. Green arrows refer to the changes in high glucose-stimulated hepatocytes under peptide intake. AKt, protein kinase B; AMPK, activated protein kinase; CREB, cAMP-response element binding protein; FoxO1, forkhead box protein O1; G6 Pase, glucose-6-phosphatase; GLUT4, glucose transporter4; GS, glycogen synthase; GSK, glycogen synthase kinase; HO-1, heme oxygenase-1; IRS-1, insulin receptor substrate 1; MAPK, mitogen-activated protein kinase; Nrf2, NF-E2-related factor 2; PEPCK, phosphoenolpyruvate carboxykinase; PGC1α, peroxisome proliferator-activated receptor gamma coactivator 1α; PI3K, phosphatidylinositide 3-kinase; ROS, reactive oxygen species; TORC2, regulated target of rapamycin complex-2.

It has been proven that elevated glycogenesis and impaired gluconeogenesis are related to the activation of IRS-1/PI3K/Akt and the AMPK signaling pathway.^185,186^ The activated insulin receptor enhances the phosphorylation of the Ser307 site in IRS-1, followed by activating the phosphorylation of PI3K/Akt, and it subsequently inactivates GSK3β by phosphorylating GSK3β on Ser9.^187,188^ The phosphorylated p-Akt upregulates GLUT4 expression and promotes GLUT4 transportation through which glucose is transported into hepatocytes.^189^ Besides, the phosphorylated p-Akt phosphorylates and inactivates forkhead box protein O1 (FoxO1), which is a member of the forkhead family of transcription factors, and then reduces the levels of PEPCK and G6Pase expressions.^190^ AMPK, an intracellular energy sensor, can trigger the phosphorylation of target rapamycin complex-2 (TORC2) and inhibit its nuclear translocation. These changes can modulate the transcription factor cAMP response element binding protein (CREB) and the CREB-dependent transcription of peroxisome proliferator-activated receptor gamma coactivator 1α (PGC1α).^191^ Subsequently, the gluconeogenic targets PEPCK and G6Pase are decreased, subsequently decreasing hepatic gluconeogenesis.^184^ To date, the mechanism of peptides increasing glycogen synthesis and glucose uptake and decreasing gluconeogenesis may be mediated by activating the IRS-1/PI3K/Akt and AMPK signal pathways (Figure 3). The peptides IAVPGEVA, IAVPTGVA, and LPYP from soy glycinin could regulate glucose metabolism and increase glucose uptake via activating GLUT1 and GLUT4 by stimulation of AKT and AMPK pathways in HepG2 cells.^91^ Similarly, the peptide IPPKKNQDKTE that originated from casein glycomacropeptide enhanced glycogen synthesis via IRS-1/PI3K/Akt and AMPK signaling pathways.^192^ A walnut-derived peptide LPLLR elevated glycogenesis by promoting the phosphorylation levels of IRS-1, PI3K, Akt, and GSK3β and expression levels of GS and GLUT4 (activation of the IRS-1/PI3K/Akt pathway), while it inhibited gluconeogenesis via facilitating Thr172 phosphorylation of AMPKα and decreasing the expression of TORC2, CREB, PGC1α, G6Pase, and PEPCK (activation of the AMPK pathway).^193^

Substantial evidence has shown that reactive oxygen species (ROS) are critical for triggering the pathogenesis of insulin resistance and T2D.^194^ The excess production of ROS aroused by overnutrition and inflammation in obesity and T2D is related to the activation of ERK, JNK, or p38 MAPK and the inhibition of the insulin response.^195^ Restraining ROS-mediated MAPK signaling pathways has been proven to alleviate insulin resistance.^196^ Interestingly, peptide IPPKKNQDKTE decreased the overproduction of ROS induced by high glucose and activation of MAPK signaling, accompanied by inhibition of the Ser307 and Ser636/639 phosphorylation of IRS-1.^197^ The serine phosphorylation, especially on Ser636/639 and Ser307, and tyrosine phosphorylation of IRS-1 protein inhibited the activity of the IR kinase and subsequently impaired insulin signaling.^198,199^ This indicates that peptides inhibit insulin resistance and increase the glucose uptake probably via inhibition of ROS-mediated MAPK signaling, subsequently inhibiting IRS-1 Ser636/639 and Ser307 phosphorylation (Figure 3). Besides, the NF-E2-related factor 2 (Nrf2)/heme oxygenase-1 (HO-1) signaling pathway is of vital importance in resisting oxidative stress and insulin resistance. The increase in Nrf2 nuclear translocation elevates HO-1 expression and modulates the ROS levels by stimulating HO-1-regulated transcription of antioxidant proteins and increasing antioxidants (glutathione and bilirubin).^200,201^ Peptide IPPKKNQDKTE was also found to activate Nrf2/HO-1 signaling, which contributes to high glucose-induced ROS production and insulin resistance.^197^ A recent study on a peptide GPPGPA from collagen hydrolysates of Chinese giant salamander skin also had an effect on alleviating insulin resistance, accompanied by promotion of the activity of ROS-scavenging superoxide dismutase (SOD).^202^

The effects of peptides on lipid metabolism and the related gene expression in HepG2 cells were also investigated. The liver assembles a very low-density lipoprotein (VLDL) particle from one apolipoprotein B-100 (apoB-100) molecule, which carries cholesteryl esters, TG, and a number of other apolipoproteins and lipids.^203^ It is known that elevated secretion of apoB-100 by the liver is a biomarker of hepatic lipid abnormality.^204^ The 7S-peptides derived from highly purified soybean β-conglycinin (7S) suppressed the secretion of apoB-100 and increased the mRNA expressions of genes related to fatty acids β-oxidation.^205^ Moreover, peptides LA, VL, and ST from soy protein isolate significantly inhibited TG synthesis and apoB secretion.^206^

### Other Cell Models

Effects of the peptides were also tested in other cell models. Skeletal muscle cells such as C2C12 myotubes and rat-derived L6 myoblasts are also used in investigating the influences of bioactive peptides on glucose metabolism and insulin resistance. Except for adipose tissue and heart muscle, skeletal muscle is the major site of insulin-stimulated glucose transport, accounting for 25 to 30% of postprandial glucose consumption.^207^ Glucose uptake in muscle is markedly decreased under insulin resistance, and this change will facilitate the systemic impairment of glucose homeostasis and hyperglycemia.^208^ Insulin resistance in skeletal muscle is related to inflammation, oxidative stress, and accumulation of intramyocellular lipids.^209−211^ Furthermore, in skeletal muscle, the tyrosine phosphorylation of IRS-1 and subsequently phosphorylation of Akt signaling promote glucose uptake. The whey peptides reduced the palmitate-induced insulin resistance, inflammation, and diacylglycerol accumulation in C2C12 myotubes.^160^ Viscothionin, with a 6-kDa basic polypeptide structure, showed hypoglycemic activity by upregulating the expression of GLUT-4, IRS-1, and AKT in differentiated C2C12 cells.^212^ In muscle L6 cells, peptide fractions isolated from soybeans enhanced the glucose uptake, mainly via the activation of the AMPK enzyme.^213^ Besides, peptide IRW derived from egg white attenuated the reduction in glucose uptake induced by Ang-II, normalized serine phosphorylation of IRS and GLUT4 expression, decreased the Ang-II receptor type 1 (AT1R) activity, and increased the p-AKT.^47^ It has been proven that the binding of Ang II with its receptor AT1R can activate nicotinamide adenine dinucleotide phosphate (NADPH) oxidase and thus promote the production of ROS.^214^ Elevation of ROS production induces the IRS serine phosphorylation and causes a decrease of GLUT4 translocation, leading to an impaired insulin signaling pathway.^215^ Peptide IRW was found to decrease ROS formation and NADPH oxidase.^47^

Rat insulinoma RINm5F cells and insulinoma INS-1E cells, from the pancreatic β-cell, are models to study the glucose-sensing function. Peptide fractions from common bean significantly promoted glucose-stimulated insulin secretion, accompanied by inhibiting DPP-IV levels and the receptor for advanced glycation end products (RAGE) and reducing the ROS in rat insulinoma INS-1E cells.^216^ The development of chronic diabetic complications is associated with the increase of RAGE in pancreatic islets, and RAGE attracts the apoptosis of the pancreatic β-cell via NADPH oxidase mediated ROS generation.^217^ Viscothionin was also found to increase insulin secretion from RINm5F cells.^212^ The mechanism involved in peptide-induced insulin secretion in insulinoma cells is not clearly understood, and further studies are needed in the future.

Taken together, the food-derived bioactive peptides could be a potential dietary supplement to help manage obesity and diabetes by intervening in the activities of enzymes, such as DDP-IV and α-glucosidase, and by modulating inflammation, lipid metabolism, and insulin resistance. However, the available data on the stability, kinetics, and bioavailability of these bioactive peptides are scarce. The methods of improving the bioavailability of bioactive peptides should be further investigated.

### In Vivo Studies of Peptides

**Figure 4:**
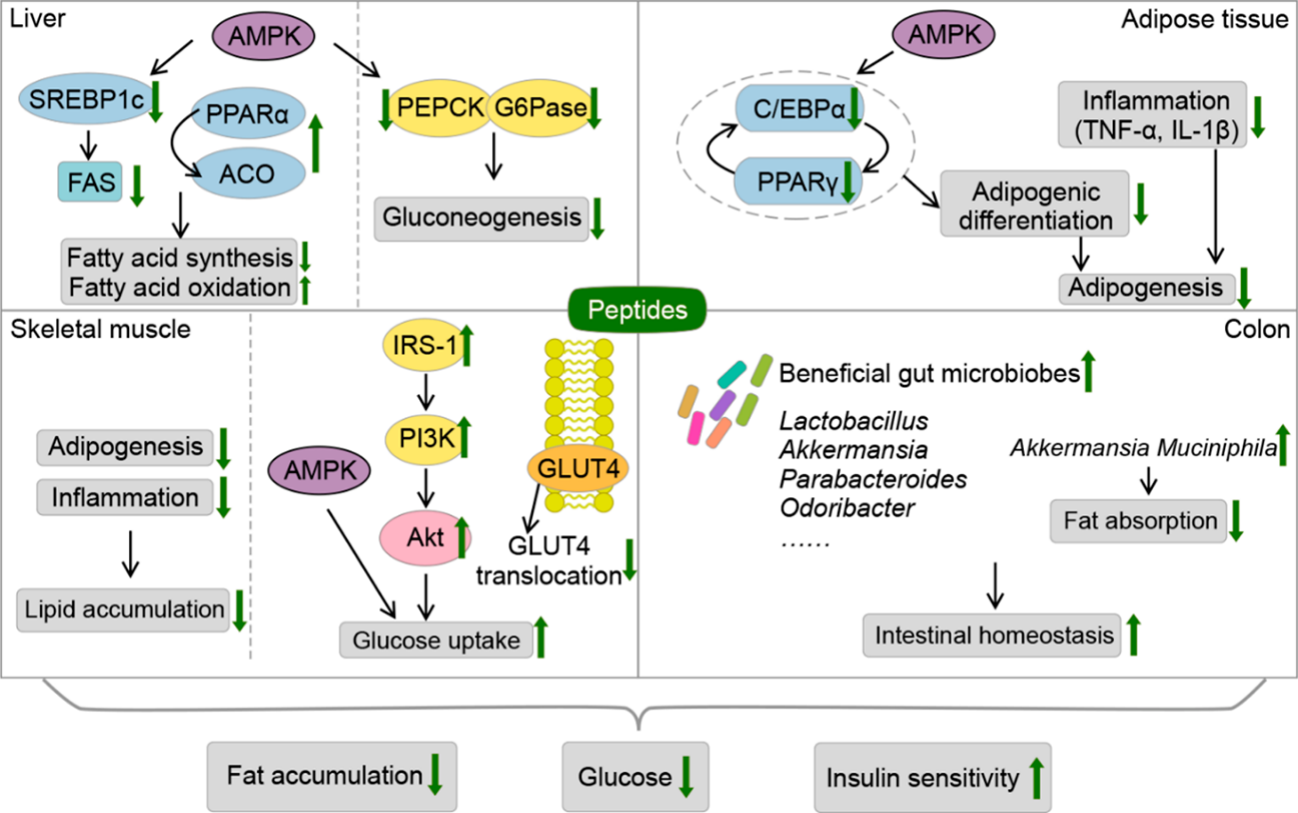
Mechanism for amelioration of obesity and diabetes by bioactive peptides in the liver, skeletal muscle, and adipose tissue. AMPK is one of the most critical regulators of lipid and glucose metabolism. The activation of AMPK by the intervention of peptides helps to regulate fatty acid metabolism and inhibit gluconeogenesis in liver, and suppress adipogenesis in adipose tissue. Peptides could improve glucose uptake via increasing the AMPK and IR/IRS-1 pathways and GLUT4 translocation in the skeletal muscle. Peptides could help to maintain the stability of intestinal homeostasis and regulate gut microbiota. ACO, acyl-CoA oxidase; AKt, protein kinase B; AMPK, activated protein kinase; C/EBP-α, CCAAT/enhancer binding protein alpha; FAS, fatty acid synthase; G6 Pase, glucose-6-phosphatase; GLUT4, glucose transporter 4; IL-1β, interleukin 1β; IRS-1, insulin receptor substrate 1; PEPCK, phosphoenolpyruvate carboxykinase; PI3K, phosphatidylinositide 3-kinase; PPAR, peroxisome proliferator activated receptor; SREBP1c, sterol regulatory element binding protein 1c; TNF-α, tumor necrosis factor alpha.

The *in vitro* studies are normally conducted to explore the underlying mechanisms of the active peptides. Studies *in vivo* can provide direct evidence that food-derived bioactive peptides are beneficial in alleviating obesity and diabetes. However, there is a concern if concentrations of most food-derived peptides utilized in *in vitro* studies are physiologically achievable in humans. In this regard, the doses of the bioactive peptides used in the animal or human studies should be clearly justified when the relevant studies are designed. The detailed information on *in vivo* studies is summarized in Table 4. Peptides that are isolated from yogurt and salmon significantly decreased the body fat and insulin resistance as well as increased glucose tolerance in C57BL/6 mice and LDLr^–/–^/ApoB^100/100^ mice, respectively.^223,224^ To be specific, the antiobese molecular mechanisms of bioactive peptides are mainly mediated by decreasing fat accumulation, modulating fatty acid metabolism, relieving inflammation, maintaining intestinal homeostasis, and regulating gut microbiota. The antidiabetic mechanisms are mainly mediated by preventing gluconeogenesis and regulating insulin sensitivity. The specific mechanisms are proposed herein below (Figure 4).

**Table 4:** *In Vivo* Studies of Bioactive Peptides and Their Effects Related to Diabetes and Obesity^a^

peptides	source	subject	treatment details	main findings/mechanism and effect	reference
Yogurt peptides	Yogurt	Male C57BL/6 mice	0.2 g/kg BW/day	↓Body fat and ↑glucose tolerance	(223)
Salmon peptides	Salmon	Obese and VitD3-deficient LDLr^–/–^/ApoB^100/100^ male mice	High-fat high-sucrose diets, with 25% of dietary proteins replaced by salmon peptides for 12 weeks	↑Energy and ↓visceral fat accumulation with an improved adipokine profile	(224)
				↓Insulin resistance	
(−)-Ternatin [d-allo-Ile^1^-l-(NMe)Ala^2^-l-(NMe)Leu^3^-l-Leu^4^-l-(NMe)Ala^5^-d-(NMe)Ala^6^-(2*R*,3*R*)-3-hydroxy-Leu^7^] and its derivative [d-Leu^7^]ternatin	*Coriolus versicolor*	Diabetic KK-A^y^ mice	Ternatin (8.5 or 17 nmol/day) or [d-Leu^7^] ternatin (68 nmol/day) via the osmotic pumps	↓Hyperglycemia	(225)
					
				↓SREBP-1c mRNA level	
YPFVV	Soybean β-conglycinin β-subunit	KK-A^y^ mice	10 mg/kg dissolved in drinking water for 5 weeks	↓Hyperglycemia, plasma and liver TG levels and liver weight	(226)
				↑Adiponectin concentration and hepatic mRNA levels of AdipoR2	
				↑mRNA of PPARα, ACO, CPT1A, and UCP2 in liver	
Kefir peptides	Kefir	HFD-fed SD rats	Orally administered at 164 mg/kg BW daily for 8 weeks	↓FAS protein and ↑p-ACC protein to block lipogenesis in liver	(227)
				↑Fatty acid oxidation by ↑protein expressions of pAMPK, PPARα, and hepatic CPT-1 in the liver	
				↓Inflammatory cytokines (TNF-α, IL-1β, and TGF-β)	
βCG peptide	Soy β-Conglycinin	Otsuka Long-Evans Tokushima fatty (OLETF) rats	The casein and sucrose in the diet were replaced with 11.43% (w/w) βCG peptide for 4 weeks	↓Abdominal white adipose tissue weight	(228)
				↓TG and cholesterol in serum and liver	
				↓Lipogenic enzyme activity of FAS and G6PDH and ↑lipolytic enzyme activity of CPT in liver	
SWFCP	Fish collagen	HFD-fed ICR mice	300 mg/kg/day for 8 weeks by gavage	↓Body weight gain	(173)
				↓TC, TG, LDL-C; ↑HDL-C	
				↓Expression of C/EBP-α, PPARγ, and aP2 in epididymal adipose tissue	
Liposome-encapsulated peptide PDBSN (GLSVADLAESIMKNL)	Synthetic peptide	HFD-fed C57BL/6J mice	Tail vein injection with 5 mg/kg and 10 mg/kg once a week for total 8 weeks	↓Weight gain and fat mass	(229)
				Improve glucose metabolism	
				Ameliorates hepatic steatosis via decreasing FFAs and TG	
				↓AMPK signaling pathway and adipogenic differentiation in visceral adipose tissue	
VPP	Milk	Diet-induced obese C57BL/6J mice	High-fat high-sucrose diet and VPP (0.1% in water) for 4 months	↓TNF-α and IL-1β expression in adipose tissue	(48)
				↑Insulin resistance	
				↓Macrophage accumulation and activation in fat tissues	
VHVV	Soybean	HFD-fed C57BL/6J mice	5, 15, 25 mg/kg/day through intraperitoneal (IP) injection for 6 weeks	↓Lipid accumulation in muscle tissues of mice	(231)
				↓TNF-α expression	
				↓Protein expressions of caspase 8 and caspase 3 related to apoptosis in soleus muscle	
				↑Proteins expressions of PPARα and Foxo3a in soleus muscle	
Protein hydrolysate	*Spirulina platensis* protein	HFD-fed C57BL/6J mice	2 g/kg BW/day for 4 weeks by gavage	↓BW, serum glucose, and TC via modulating expressions of Acadm, RETN, Fabp4, Ppard, and Slc27a1 in the brain and liver	(232)
Collagen peptide with 10.30% hydroxyproline and 8.99% proline	Walleye pollock skin	HFD-fed C57BL/6J male mice	Peptide was mixed into the daily drinking water with a volume of 800 mg/kg/day for 8 weeks	↓Weight gain, adipose mass in liver and adipocytes	(233)
				↓TG, TC, HDL-c, LDL-c, and FFA	
				↑ Relative abundances of Lactobacillus, Akkermansia, Parabacteroides, and Odoribacter spp	
				↓Abundances of Erysipelatoclostridium and Alistipes	
A 9-amino-acid peptide named D3	Human α-defensin 5	HFD-fed C57BL/6J mice, Sprague–Dawley (SD) rats	NA	↓Body weight	(234)
				Ameliorated leptin resistance	
				↓Appetite via the UGN-GUCY2C endocrine axis	
				↑The abundance of intestinal *Akkermansia muciniphila* through the IFNγ-Irgm1 axis	
DIKTNKPVIF	Potato protein	STZ-induced diabetic mice	25 and 50 mg/kg/day for 4 weeks by oral gavage	↓Blood glucose	(235)
				↓Plasma total glycerol, TC, insulin, and HbA_1c_	
				↑The population of β-cells expressing insulin	
				↓Inflammation	
Peptides	*Palmaria palmata*	STZ-induced diabetic mice	50 mg/kg bodyweight by oral gavage twice-daily for 18 days	Exerted antidiabetic effects via ↓blood glucose and ↑insulin	(236)
				↑Terminal oral glucose tolerance and fasting blood glucose	
Aglycin (ASCNGVCSPFEMPPCGSSACRCIPVGLVVGYCRHPSG)	Soy	HFD and STZ treated T2D BALB/c mice	50 mg/kg orally daily for 28 days	Ameliorated glucose intolerance and insulin resistance	(92)
				↑Glucose utilization and insulin sensitivity	
				↑Glucose uptake	
				↑Expressions of p-IR, p-IRS1, p-Akt, and membrane GLUT4 protein in the skeletal muscle	
Sea cucumber hydrolysates with 242 peptides	Sea cucumber	HFD and STZ-induced diabetic rats	200 and 400 mg/kg BW	Attenuated body weight loss	(237)
				Improved glucose tolerance	
				↑Expressions of PI3K, p-Akt, p-GSK-3β, and GLUT2/4 pathways	
				↓Expression of p-IRS1 in liver and skeletal muscle	
Marine collagen peptides	Fish	Chinese T2D patients	13 g daily for 3 months	↓Fasting blood glucose, human GHbA1c, fasting blood insulin, TG, TC, LDL, and free-fatty acids	(238)
				↑Levels of insulin sensitivity index and HDL	
Oligopeptide	β-casein hydrolysate	Obese Patients	Daily intake of a β-casein-hydrolysate-whey drink for 8 weeks	↑FGF-21	(239)
Enzymatic hydrolysate	*Styela clava* flesh tissue hydrolysate	Patients with diabetes	500 mg/day daily for 4 weeks	↓Hemoglobin A1c and plasma insulin levels	(240)
A cod protein hydrolysate	Atlantic cod (*Gadus morhua*) meat	41 healthy individuals	20 mg/kg body weight given before a breakfast meal	↓Postprandial insulin	(241)
Protein Hydrolysate peptides	Milk whey protein	Prediabetic subjects	1400 or 2800 mg/kg BW for 6 weeks	↓Glucose iAUC and expressed a minor insulinotropic effect	(242)
				↓HbA_1c_ values	

a Abbreviations: Acadm, acyl-CoA dehydrogenase medium chain; ACC, acetyl-CoA carboxylase; p-ACC, p-acetyl-CoA carboxylase; AKT, protein kinase B; BW, body weight; CPT-1, carnitine palmitoyltransferase-1; Fabp4, fatty acid-binding protein 4; FBG, fasting blood glucose; GHb, glycated hemoglobin; FGF21, fibroblast growth factor 21; GLP-1, glucagon-like peptide-1; GLUT2, glucose transporter2; GLUT4, glucose transporter4; GSK3β, glycogen synthase kinase 3β; HbA_1c_, glycated hemoglobin A1c; HDL, high-density lipoprotein; HDL-C, high-density lipoprotein cholesterol; HFD, high fat diet; HOMA-IR, homeostasis model assessment of insulin resistance; IFN-γ, interferon gamma; IL-1β, interleukin 1β; IRS-1, insulin receptor substrate 1; ISI, insulin Sensitivity index; LDL-C, low-density lipoprotein cholesterol; MCP-1, monocyte chemoattractant protein-1; MW, molecular weight; NA, not available; OGTT, oral glucose tolerance test; Ppard, peroxisome proliferator activated receptor delta; PPARγ, peroxisome proliferator associated receptor gamma; RETN, resistin; Slc27a1, solute carrier family 27 member 1; T2D, diabetes mellitus type 2; TC, total cholesterol; TG, triglyceride; TGF-β, transforming growth factor-β; TNF-α, tumor necrosis factor alpha; UCP-2, uncoupling protein-2.

### Anti-Obesity

The peptides could prevent fat accumulation via decreasing fat synthesis and promoting fatty acid oxidation in the liver. A mushroom-derived cyclic peptide called ternatin could suppress hyperglycemia and fatty acid synthesis by decreasing mRNA levels of SREBP-1c in KK-A^y^ mice administered with 8.5 or 17 nmol/day ternatin via osmotic pumps.^225^ Supplementation with 10 mg/kg soymorphin-5 in drinking water for 5 weeks could decrease the TG levels in KK-A^y^ mice through activation of adiponectin and mRNA expressions of PPARα, ACO, CPT1A, and UCP2.^226^ Kefir peptides had also been shown to block lipogenesis via reducing FAS and promoting fatty acid oxidation via increasing the gene expressions of pAMPK, PPARα, and CPT-1 in SD rats given 164 mg/kg BW for 8 weeks.^227^ Similarly, soy β-conglycinin peptides could decrease the activities of lipogenic enzymes FAS and G6PDH and increase the activities of lipolytic enzyme CPT-1 in obese rats.^228^

Research has shown that peptides could ameliorate adipogenic differentiation and adipogenesis in adipose tissue *in vivo*. Fish collagen peptide could reduce adipogenesis via modulating adipogenic regulators C/EBP-α and PPARγ in adipose tissue when HFD-fed mice were gavaged with this peptide at a dose of 300 mg/kg/day for 8 weeks.^173^ In contrast, it also controlled lipid accumulation in 3T3-L1 preadipocytes at a concentration of 1.0 mg/mL.^173^ A liposome-encapsulated peptide, PDBSN was prepared to improve stability and specificity. It was found that supplementation of 5–10 mg/kg BW PDBSN in HFD-fed mice significantly restricted adipogenic differentiation in adipose tissue via activating the AMPK signaling pathway.^229^ Milk-derived peptide VPP is one of the popular food-derived bioactive peptides, mainly due to its ACE-inhibiting activities. The bioavailability of VPP detected in a pig model has been estimated to be around 0.1%, and the half-life was 15 min.^230^ It was found that drinking 0.01% VPP water for 4 months could suppress adipose inflammation with downregulation of expression of TNF-α and IL-1β and a decrease in accumulation of macrophages in fat tissues.^48^

Skeletal muscle plays a major role in fatty acid uptake and oxidation and is closely related to the occurrence of obesity. It was found that the soybean peptide VHVV alleviated obesity-related muscle wasting through inhibiting TNF-α expression and lipid accumulation, improving apoptosis, and reducing muscle loss in skeletal muscle of C57BL/6J mice.^231^

It has been determined that some bioactive peptides have beneficial effects on the gut-brain-liver axis via regulation of gut microbiota. Zhao et al. prepared *Spirulina platensis* protein hydrolysate, which contained peptides with a low molecular weight.^232^ Oral intake of these peptides at a high dosage of 2 g/kg BW/day in mice expressed antiobesity activity by modulating the expressions of acyl-CoA dehydrogenase medium chain (Acadm), resistin (RETN), fatty acid-binding protein 4 (Fabp4), peroxisome proliferator activated receptor delta (Ppard), and solute carrier family 27 member 1 (Slc27a1) in the gut-brain-liver axis.^232^ Furthermore, supplementation of collagen peptide from Walleye pollock skin at 800 mg/kg BW/day in obese mice for 8 weeks could favorably modulate the gut microbiota with increasing the relative abundance of Lactobacillus, Akkermansia, Parabacteroides, and Odoribacter spp. and decreasing that of intestinal inflammation bacteria, such as Erysipelatoclostridium and Alistipes.^233^ A recent study also proved that a nine-amino-acid peptide prevented obesity by suppressing appetite via the UGN-GUCY2C endocrine axis and modulating gut microbiota.^234^ This peptide significantly raised the abundance of *A. muciniphila*, which may further suppress fat absorption.

### Anti-Diabetes

Peptides from potato protein and *Palmaria palmata* showed antidiabetic activity mediated by regulating blood glucose and insulin changes in streptozotocin (STZ)-induced diabetic mice.^235,236^ Aglycin with 37 amino acids originated from soy could improve glucose tolerance and insulin resistance via increasing the IR/IRS1 insulin receptor signaling pathway and GLUT4 translocation in the skeletal muscle of T2D mice.^92^ It was found that these peptides at 50 mg/kg of BW could help to alleviate diabetes. Besides, sea cucumber hydrolysates could improve insulin resistance via activating the PI3K/Akt pathway in diabetic rats at 200–400 mg/kg.^237^ The analysis of UPLC-qTOF-MS/MS found that this hydrolysate was a mixture of peptides of 242 kinds of peptides. These results suggested that dietary bioactive peptides were beneficial in the management of diabetes via modulating the IRS-1/PI3K/Akt and AMPK signaling pathways.

### Clinical Studies

There have been only a few clinical studies to test the effect of bioactive peptides on obesity and diabetes. One study demonstrated that Chinese T2D patients, who received 13 g of marine collagen peptides each day for 3 months, showed improved glucose hemostasis and increase insulin sensitivity with a decrease in fasting insulin by 19.8%. with an increase in the insulin sensitivity index by 20%.^238^ In obese patients, oral supplementation of 0.5 g of β-casein hydrolysate in whey drink for 8 weeks could increase the serum concentration of fibroblast growth factor 21 (FGF-21), a metabolic regulator in lowering glucose and improving insulin sensitivity.^239^ In addition, several studies showed that protein hydrolysates with high amounts of peptides could also exhibit an antidiabetic effect. Consumption of 500 mg enzymatic hydrolysate from *Styela clava* flesh tissue hydrolysate daily for 4 weeks significantly decreased glycated hemoglobin (HbA_1C_) levels by 2.7% (*p* < 0.05) compared with the basal level in diabetic patients.^240^ In a double-blind crossover trial, a low dose at 20 mg/kg BW of marine protein hydrolysate from Atlantic cod (*Gadus morhua*) was given to diabetic subjects. It was found that postprandial insulin levels were significantly decreased.^241^ The authors proposed that some branched-chain amino acid-containing peptides in the hydrolysate were absorbed via peptide transporters in the upper jejunum and then into the blood, and they exerted a unique bioactive effect, although the concentration was low.^241^ Another randomized crossover trial demonstrated that hydrolyzed peptides derived from milk whey protein could reduce the postprandial glucose incremental areas under the curve (iAUC) by 20.2% after a single dose (1400 mg bioactive peptides) intake and decrease glycated hemoglobin (HbA_1C_) levels by 10.5% after 6 weeks of intervention in prediabetic subjects.^242^ However, the bioavailability and metabolic fate of these bioactive peptides were not clear. Quantitatively and qualitatively, randomized, double-blind, controlled clinical trials are needed to validate the effects of these bioactive peptides in the management of obesity and diabetes in humans.

## Future Perspectives

In summary, this paper summarized the top-down and bottom-up production methods, as well as the bioavailability of food-derived bioactive peptides, and reviewed the recent *in vitro*, *in vivo*, and clinical studies concerning their possible benefits as a kind of dietary supplement in management of obesity and diabetes. The studies in cell lines and animal models clearly demonstrated that bioactive peptides were beneficial in modulating the metabolism of glucose and lipids through several molecular mechanisms, including inhibiting the activities of relative enzymes related to lipogenesis, and regulating insulin sensitivity mediated by their effects on PPAR, and AMPK pathways. It should be pointed out that the doses of bioactive peptides used in some animal studies are too high to be achieved under the normal physiological conditions in humans. The translation and extrapolation of such data into clinical application require further investigation. On the one hand, factors affecting the permeability, metabolism, absorption, gastrointestinal degradation, bioavailability, and pharmacodynamics of bioactive peptides remain to be fully studied. Although several clinical studies have demonstrated that bioactive peptides are beneficial in the management of diabetes, more double-blind randomized controlled trials in a large scale are needed in the future. On the other hand, it is necessary to explore more administration forms to increase the bioavailability of bioactive peptides. To improve the bioavailability and potency of bioactive peptides, the following measures can be considered, including (1) structure modifications to protect the peptides from gastrointestinal degradation, (2) site-specific delivery of peptides to extend their efficacy and duration of action, and (3) application of absorption enhancers to promote the uptake of peptides into blood circulation.^136,139^ Furthermore, the structure–function and composition-function relationships between the bioactive peptides and their antiobesity and antidiabetic properties should be further investigated. In view that the prevalence of DM and obesity is more severe in the world, food-derived bioactive peptides have the potential to be developed as functional foods or nutraceuticals in the management of obesity and diabetes.
